# First-line immune checkpoint inhibitors plus targeted therapy versus sorafenib or lenvatinib monotherapy for unresectable or advanced hepatocellular carcinoma: a meta-analysis of phase 3 trials

**DOI:** 10.3389/fimmu.2025.1667793

**Published:** 2025-10-15

**Authors:** Yuxuan Lin, Yonghe Liao, Bo Luo, Jinhai Shen

**Affiliations:** ^1^ Department of Pharmacy, Guangxi Hospital Division of The First Affiliated Hospital, Sun Yat-Sen University, Nanning, Guangxi, China; ^2^ School of Pharmaceutical Science, Guangxi Medical University, Nanning, Guangxi, China; ^3^ Department of Pharmacy, The Second Affiliated Hospital of Guangxi Medical University, Nanning, Guangxi, China; ^4^ State Key Laboratory of Natural Medicines, China Pharmaceutical University, Nanjing, Jiangsu, China; ^5^ School of Basic Medicine and Clinical Pharmacy, China Pharmaceutical University, Nanjing, Jiangsu, China

**Keywords:** immune checkpoint inhibitors, targeted therapy, sorafenib, lenvatinib, hepatocellular carcinoma, meta-analysis

## Abstract

**Background:**

Immune checkpoint inhibitor (ICI) and targeted therapy (TT) combinations have emerged as promising first-line treatments for unresectable or advanced hepatocellular carcinoma (u/aHCC), leveraging synergistic anti-tumor effects. However, the comparative efficacy and safety of ICI-TT regimens *versus* sorafenib or lenvatinib (S/L) monotherapy require further elucidation across larger patient populations. This meta-analysis synthesizes data from phase 3 trials to evaluate the clinical benefits and risks of first-line ICI-TT combination therapy in u/aHCC.

**Methods:**

We conducted systematic searches in PubMed and major oncology conference proceedings up to June 10, 2025. Eligible studies were randomized phase 3 trials comparing first-line ICI-TT *versus* S/L monotherapy in u/aHCC. Efficacy outcomes included progression-free survival (PFS), overall survival (OS) (summarized as hazard ratios [HRs] with 95% confidence intervals [CIs]), and objective response rate (ORR) (evaluated using odds ratios [ORs]). Safety outcomes assessed grade 3–5 treatment-related adverse events (TRAEs) and serious TRAEs, reported as relative risks (RRs).

**Results:**

Eight phase 3 trials (IMbrave150, ORIENT-32, COSMIC-312, CARES-310, LEAP-002, SCT-I10A-C301, HEPATORCH, APOLLO) involving 4,379 patients were included. Compared with S/L monotherapy, ICI-TT combination therapy demonstrated significantly improved ORR (OR 3.93; 95% CI 2.64–5.85), PFS (HR 0.62; 95% CI 0.54–0.71), and OS (HR 0.71; 95% CI 0.62–0.82). The risk of grade 3–5 TRAEs was not significantly increased with combination therapy (RR 1.13; 95% CI 0.96–1.33). However, combination therapy was associated with a significantly higher risk of serious TRAEs (RR 1.97; 95% CI 1.50–2.60).

**Conclusion:**

First-line ICI-plus-TT combination therapy demonstrates superior efficacy in ORR, PFS, and OS compared to S/L monotherapy for u/aHCC, without a significant increase in grade 3–5 TRAEs. Clinicians should be aware of the elevated risk of serious TRAEs associated with combination regimens. These findings support ICI-TT as a preferred first-line strategy for eligible patients, although individualized risk-benefit assessment remains crucial.

**Systematic review registration:**

https://www.crd.york.ac.uk/prospero/, identifier CRD420251053588.

## Introduction

Hepatocellular carcinoma (HCC) is the most common primary malignancy of the liver and ranks among the leading causes of cancer-related mortality worldwide (1, 2). It typically arises in the setting of chronic liver disease, including hepatitis B or C virus (HBV/HCV) infection, alcoholic liver disease, and non-alcoholic fatty liver disease, frequently on a background of cirrhosis (1). Despite advances in early detection and the development of locoregional therapies, a substantial proportion of patients are diagnosed at an unresectable or advanced stage, for which systemic therapy remains the mainstay of treatment (1, 3–5).

Historically, the multi-kinase inhibitors sorafenib and lenvatinib constituted the standard first-line therapies for unresectable or advanced HCC (u/aHCC), providing modest survival benefits with median overall survival (OS) ranging from 12 to 15 months (6, 7). However, prognosis remained poor and durable responses were uncommon.

A major paradigm shift occurred with the advent of immune checkpoint inhibitor (ICI)-based combination therapies, particularly those co-targeting the programmed cell death protein 1/programmed death-ligand 1 (PD-1/PD-L1) axis and vascular endothelial growth factor (VEGF) signaling. The landmark IMbrave150 trial was the first to demonstrate a significant OS benefit with the combination of atezolizumab and bevacizumab compared to sorafenib, achieving a median OS of 19.2 months and a 34% reduction in the risk of death (8, 9). This established a new standard of care and catalyzed the development of various ICI-based therapeutic strategies.

Subsequent phase 3 trials, however, have yielded mixed results. Studies such as ORIENT-32 (sintilimab plus bevacizumab biosimilar) (10), CARES-310 (camrelizumab plus apatinib) (11), SCT-I10A-C301 (Finotonlimab plus bevacizumab biosimilar) (12, 13), APOLLO (penpulimab plus anlotinib) (14), and HEPATORCH (toripalimab plus bevacizumab) (15) have all reported significant survival benefits, reinforcing the potential of dual ICI and VEGF blockade. Conversely, trials such as COSMIC-312 (atezolizumab plus cabozantinib) and LEAP-002 (pembrolizumab plus lenvatinib) failed to meet their primary endpoints for progression-free survival (PFS) and/or OS (16, 17). Additionally, many of these individual trials were not sufficiently powered to evaluate treatment effects in clinically relevant subgroups.

Several systematic reviews and meta-analyses have previously addressed the efficacy of ICI-based combinations in advanced HCC (18–23). These studies reinforced the paradigm shift initiated by IMbrave150, but several important gaps remain. None of the existing meta-analyses integrated the most up-to-date phase 3 trials (e.g., APOLLO, HEPATORCH, SCT-I10A-C301), nor did they provide comprehensive subgroup analyses across sex, etiology, tumor burden, AFP, ECOG status, and prior local therapy.

To address these gaps, we conducted the largest and most current meta-analysis to date, synthesizing phase 3 randomized controlled trials (RCTs) comparing ICI plus targeted therapy (TT) combinations versus sorafenib or lenvatinib (S/L) monotherapy in patients with u/aHCC. The primary aims of this study were to provide an updated comprehensive evaluation of the efficacy and safety of first-line ICI-TT combination therapy and to quantify the magnitude of benefit across different clinically relevant subpopulations, thereby informing clinical decision-making and guiding future therapeutic development.

## Methods

### Protocol and reporting guidelines

This research protocol was registered with the International Prospective Register of Systematic Reviews (PROSPERO; registration number: CRD420251053588) and was conducted in accordance with the Preferred Reporting Items for Systematic Reviews and Meta-Analyses (PRISMA) 2020 guidelines (24).

### Information sources and search strategy

A systematic search of PubMed and major oncology conference proceedings was conducted to comprehensively identify all relevant phase 3 trials published up to June 10, 2025. Search terms included 'immune checkpoint inhibitors,' 'targeted therapy,' 'hepatocellular carcinoma,' as well as the names of specific therapeutic agents. The complete search strategies are detailed in **Supplementary Table 1**.

### Selection criteria

To be included in the meta-analysis, studies were required to meet the following criteria: (i) phase 3 RCTs comparing a combination of ICI and TT with S/L monotherapy; (ii) enrollment of patients with previously untreated u/aHCC; and (iii) availability of survival outcome data, including hazard ratios (HRs) with corresponding 95% confidence intervals (CIs). Studies were excluded if they: (i) were not phase 3 RCTs; (ii) did not use S/L as the control arm; (iii) included ICI monotherapy or did not incorporate an ICI in the experimental arm; or (iv) were ongoing trials without published results at the time of the literature search. Only studies that met all inclusion criteria were incorporated into the meta-analysis.

### Data collection and assessment of risk of bias

Data from all included studies were extracted and summarized by one investigator and independently verified by a second reviewer. Where available, the following information was collected: trial name, year of publication, sample size, treatment regimens, HRs with corresponding 95% CIs for PFS and OS, the number of patients who achieved complete and partial responses, and the incidence of grade 3–5 and severe treatment-related adverse events (TRAEs). Grade 3–5 TRAEs refer to the severity of adverse events as graded by the Common Terminology Criteria for Adverse Events (CTCAE). Grade 3 events are severe, Grade 4 are life-threatening, and Grade 5 are fatal. Serious TRAEs refer to the medical significance of the event, regardless of its CTCAE grade. A serious TRAE is defined by regulatory criteria as any event that results in death, is life-threatening, requires inpatient hospitalization or prolongation of existing hospitalization, results in persistent or significant disability/incapacity, or is a congenital anomaly/birth defect. In addition, study design characteristics were collected to assess the risk of bias for each included trial. The risk of bias was evaluated in accordance with the Cochrane Risk of Bias Assessment Tool (25).

### Statistical analysis

Pooled estimates were generated using either fixed-effects or random-effects models, depending on the degree of heterogeneity observed. Statistical heterogeneity was assessed using the *I²* statistic and Cochrane’s *Q* test, with heterogeneity considered significant when *I²* exceeded 50% and the *Q* test *p*-value was less than 0.1. Specifically, DerSimonian-Laird random-effects models were employed for outcomes with substantial variability, whereas Mantel-Haenszel fixed-effects models were applied when homogeneity was strongly supported by the data.

To evaluate therapeutic efficacy, pooled HRs with corresponding 95% CIs were calculated for PFS and OS, while odds ratios (ORs) with 95% CIs were computed for objective response rate (ORR). For safety assessments, relative risks (RRs) with 95% CIs for AEs were determined on a per-study basis to provide a comprehensive evaluation. Given the limited statistical power to definitively rule out heterogeneity in subgroup analyses, random-effects models were applied in these analyses.

Potential publication bias was examined using funnel plots and Egger’s test. Sensitivity analyses were performed using a leave-one-out approach to evaluate the robustness of the pooled estimates. All statistical analyses were conducted using R software (version 4.5.1), with a two-tailed *p*-value of < 0.05 considered indicative of statistical significance.

## Results

### Eight phase 3 trials with 4,379 patients were included

The literature search identified 64 records, of which eight trials met the eligibility criteria and were included in the final analysis (8–12, 14–17, 26). The PRISMA flow diagram illustrating the study selection process is presented in **Supplementary Figure 1**.

Among the eight included trials, all employed an open-label design. A total of 4,379 patients with u/aHCC were enrolled, of whom 2,640 (60.3%) received a combination of ICIs and TT, while 1,739 (39.7%) received S/L monotherapy. The ICI used in the experimental arms included atezolizumab (anti–PD-L1), sintilimab, camrelizumab, pembrolizumab, SCT-I10A, toripalimab, and penpulimab (all anti–PD-1 agents). The targeted therapies administered in the combination regimens comprised bevacizumab, bevacizumab biosimilars (IBI305 and SCT5101), cabozantinib, rivoceranib, lenvatinib, and anlotinib. In all trials, the control arms consisted of S/L monotherapy. Detailed characteristics of each trial are summarized in **Table 1**. Thus, the analysis included a substantial cohort of patients from globally conducted trials, providing a robust foundation for the subsequent efficacy and safety evaluations.

**Table 1:** Main characteristics of the included phase 3 trials.

Study	Year	Design (randomization)	Sample size	Drugs used in experimental arm	Targets of targeted therapy	Drugs used in control arm	Population characteristics	mPFS (mon) HR for PFS (95% CI)	mOS (mon) HR for OS (95% CI)
IMbrave150 (8, 9) (NCT03434379)	2020 2022	Phase 3, open label RCT (2: 1)	501 Exp: 336 Ctrl: 165	Atezolizumab and bevacizumab	VEGF-A	Sorafenib	Treatment-naive adults with unresectable HCC, aged ≥ 18 years, 83% male. Enrolled in 17 countries (40% in Asia excluding Japan). 69% had HBV or HCV, macrovascular invasion/extrahepatic spread (75%), Child-Pugh A (99%), BCLC stage C (82%), and α-fetoprotein ≥ 400 ng/mL (37%).	6.9 *vs* 4.3 0.65 (0.53–0.81)	19.2 *vs* 13.4 0.66 (0.52–0.85)
ORIENT-32 (10) (NCT03794440)	2021	Phase 2-3, open label RCT (2: 1)	571 Exp: 380 Ctrl: 191	Sintilimab plus bevacizumab biosimilar (IBI305)	VEGF-A	Sorafenib	Treatment-naive adults with unresectable HCC, aged ≥18 years, 88% male. Enrolled in China. 94% had HBV, macrovascular invasion/extrahepatic metastasis (79%), Child-Pugh A (96%), BCLC stage C (85%), α-fetoprotein ≥ 400 ng/mL (43%), and previous local therapy (82%).	4.6 *vs* 2.8 0.56 (0.46–0.70)	NR *vs* 10.4 0.57 (0.43–0.75)
COSMIC-312 (16, 26) (NCT03755791)	2022 2024	Phase 3, open label RCT (2: 1)	649 Exp: 432 Ctrl: 217	Atezolizumab and cabozantinib	VEGFR2, MET, AXL, RET	Sorafenib	Treatment-naive adults with advanced HCC aged ≥ 18, 84% male. Enrolled across 32 countries (28% Asian). 61% had HBV and/or HCV, 69% extrahepatic spread/macrovascular invasion, BCLC stage B (33%) or C (67%), Child-Pugh A (99.8%), and α-fetoprotein ≥ 400 ng/mL (35%).	6.9 *vs* 4.3 0.74 (0.56–0.97)	16.5 *vs* 15.5 0.98 (0.78–1.24)
CARES-310 (11) (NCT03764293)	2023	Phase 3, open label RCT (1: 1)	543 Exp: 272 Ctrl: 271	Camrelizumab and rivoceranib	VEGFR2	Sorafenib	Treatment-naive patients with unresectable HCC, aged ≥ 18, 84% male. Conducted across 13 countries, 83% Asian. 75% had HBV, macrovascular invasion/metastasis (74%), Child-Pugh A (100%), ECOG 0/1. 36% had α-fetoprotein ≥ 400 ng/mL, 86% BCLC stage C. 57% received prior local therapy.	5.6 *vs* 3.7 0.52 (0.41–0.65)	22.1 *vs* 15.2 0.62 (0.49–0.80)
LEAP-002 (17) (NCT03713593)	2023	Phase 3, open label RCT (1: 1)	794 Exp: 395 Ctrl: 399	Pembrolizumab and lenvatinib	VEGFR1-3, FGFR1-4, PDGFRα, RET, KIT	Lenvatinib and placebo	Treatment-naive adults with unresectable HCC, aged ≥ 18 years, 53% > 65 years, 81% male. Enrolled globally (43% Asian, 43% White). 63% had viral etiology, macrovascular invasion or extrahepatic spread (67%), Child-Pugh A (99%), BCLC stage C (77%), and α-fetoprotein > 400 ng/mL (32%).	8.2 *vs* 8.1 0.81 (0.69–0.95)	21.1 *vs* 19.0 0.84 (0.71–0.98)
SCT-I10A-C301 (12, 13) (NCT04560894)	2024 2025	Phase 3, open label RCT (2: 1)	346 Exp: 230 Ctrl: 116	Finotonlimab and bevacizumab biosimilar (SCT5101)	VEGF-A	Sorafenib	Treatment-naive adults with advanced HCC in China, aged ≥ 18, 87% male. 89% had HBV, BCLC stage C (80%), α-fetoprotein ≥ 400 ng/mL (47%), macrovascular invasion (39%) or extrahepatic metastasis (61%), Child-Pugh A (93%) or B (≤ 7).	7.1 *vs* 2.9 0.50 (0.38–0.65)	22.1 *vs* 14.2 0.60 (0.44–0.81)
HEPATORCH (15) (NCT04723004)	2025	Phase 3, open label RCT (1: 1)	326 Exp: 162 Ctrl: 164	Toripalimab and bevacizumab	VEGF-A	Sorafenib	Treatment-naive adults with advanced HCC, aged 18–75 years, 87% male. Enrolled mainly in mainland China (96%). 90% had HBV, macrovascular invasion/extrahepatic spread (79%), Child-Pugh A (100%), BCLC stage C (79%), α-fetoprotein ≥ 400 ng/mL (47%), and previous local therapy (50%).	5.8 *vs* 4.0 0.69 (0.53–0.91)	20.0 *vs* 14.5 0.76 (0.58–0.99)
APOLLO (14) (NCT04344158)	2025	Phase 3, open label RCT (2: 1)	649 Exp: 433 Ctrl: 216	Penpulimab and anlotinib	VEGFR2/3, FGFR1-4, PDGFRα/β, c-KIT, RET	Sorafenib	Treatment-naive Chinese patients with unresectable HCC, aged 18–75 years, 85% male. 84% had HBV infection, macrovascular invasion or extrahepatic metastasis (80%), Child-Pugh A (93%) or B (≤ 7), BCLC stage B or C, and ECOG 0/1. 49% had α-fetoprotein ≥ 400 ng/mL.	6.9 *vs* 2.8 0.52 (0.41–0.66)	16.5 *vs* 13.2 0.69 (0.55–0.87)

RCT, randomized controlled trial; Exp, experimental arm; Ctrl, control arm; HBV, Hepatitis B virus; HCV, Hepatitis C virus; BCLC, Barcelona Clinic Liver Cancer. ECOG, Eastern Cooperative Oncology Group; mPFS, median progression-free survival; mOS, median overall survival; HR, hazard ratio; CI, confidence interval.

### ICI-TT combination therapy significantly improved ORR, PFS, and OS

**Figure 1:**
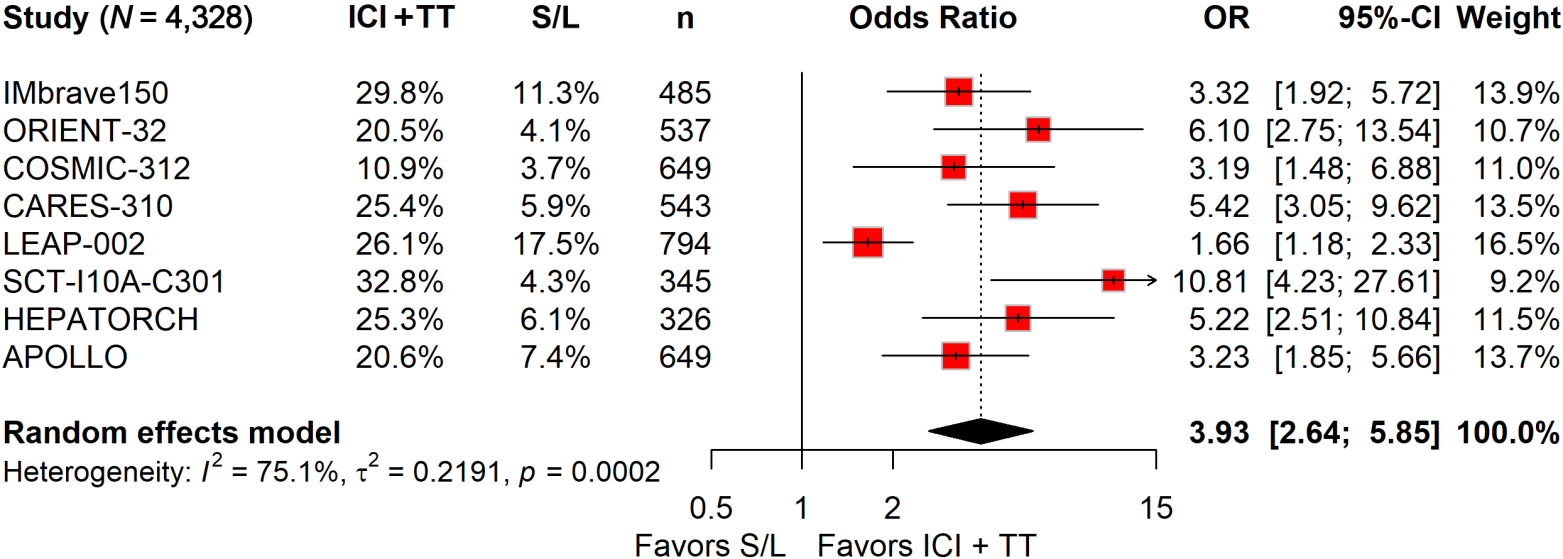
Forest plot of objective response rate comparing immune checkpoint inhibitors plus targeted therapy *versus* sorafenib or lenvatinib monotherapy in patients with hepatocellular carcinoma.

**Figure 2:**
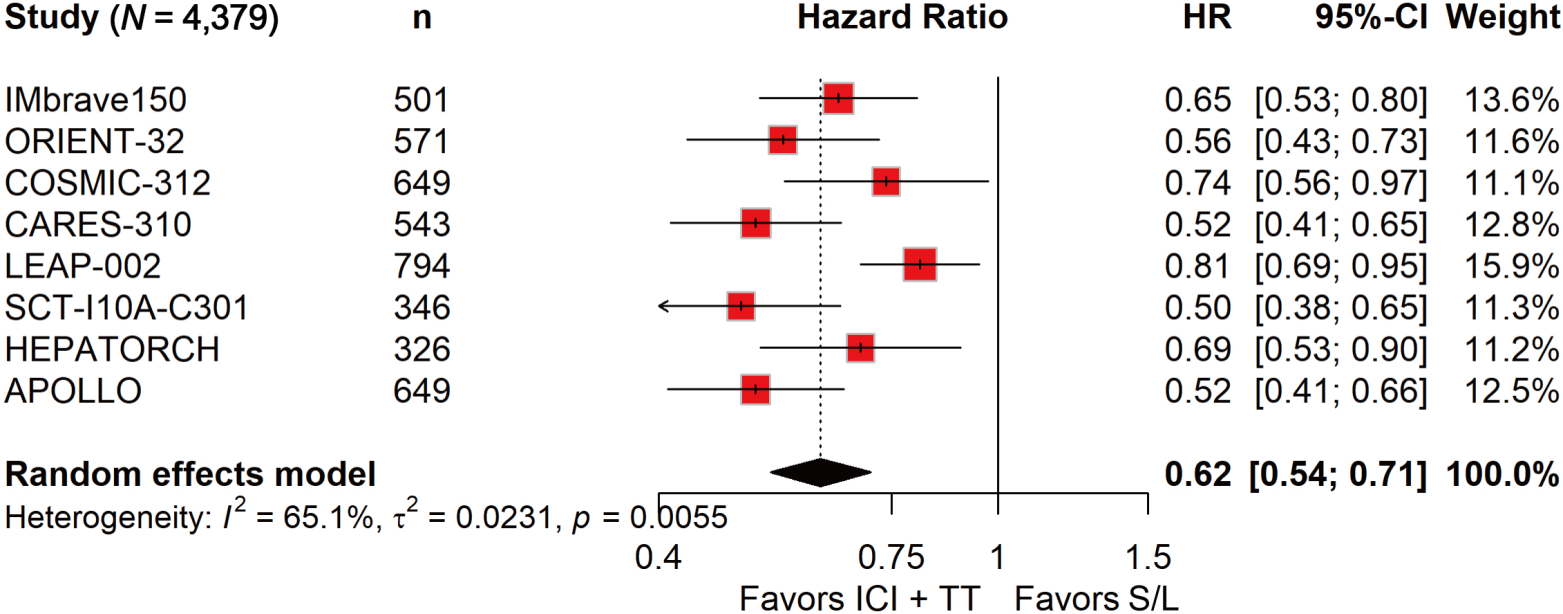
Forest plot of progression-free survival comparing immune checkpoint inhibitors plus targeted therapy *versus* sorafenib or lenvatinib monotherapy in patients with hepatocellular carcinoma.

**Figure 3:**
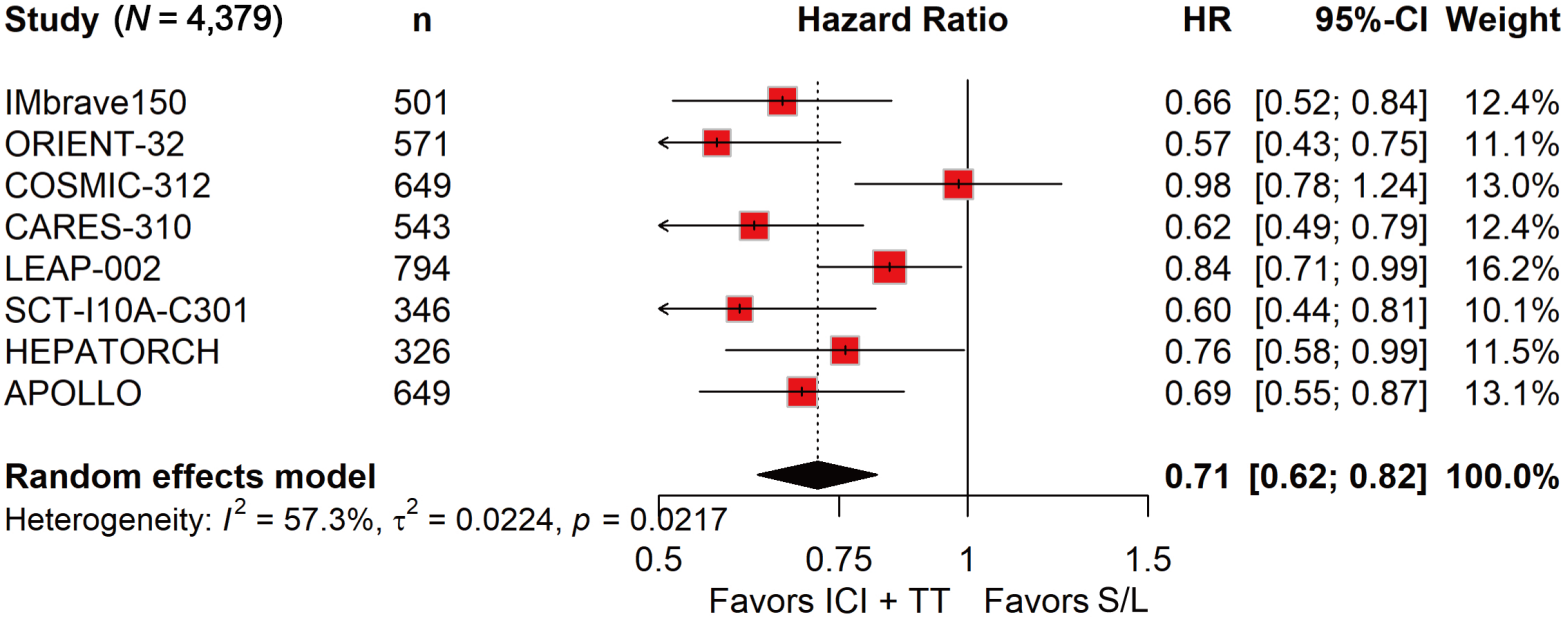
Forest plot of overall survival comparing immune checkpoint inhibitors plus targeted therapy *versus* sorafenib or lenvatinib monotherapy in patients with hepatocellular carcinoma.

ORR data, derived from 4,328 patients, showed a nearly fourfold increase in response with ICI-TT compared to S/L monotherapy (OR, 3.93; 95% CI, 2.64–5.85; **Figure 1**). These data indicate that ICI-TT combination therapy markedly enhances the tumor response rate compared to S/L monotherapy. PFS and OS data were available from all eight trials, encompassing a total of 4,379 patients. The pooled HR for PFS demonstrated a significant benefit with ICI-TT compared to S/L monotherapy (HR, 0.62; 95% CI, 0.54–0.71; **Figure 2**), corresponding to a 38% relative reduction in the risk of disease progression. This represents a statistically significant and clinically meaningful reduction in the risk of disease progression. For OS, the combined HR indicated a 29% reduction in the risk of death with ICI-TT (HR, 0.71; 95% CI, 0.62–0.82; **Figure 3**). The consistency in the direction and magnitude of benefit across response and survival endpoints strongly supports the superior efficacy of ICI-TT combinations. Collectively, these results demonstrate that first-line ICI-TT therapy provides substantial and consistent improvements in both tumor control and overall survival for patients with u/aHCC.

### The risk of serious TRAEs was higher with ICI-TT, while grade 3–5 TRAEs were comparable

**Figure 4:**
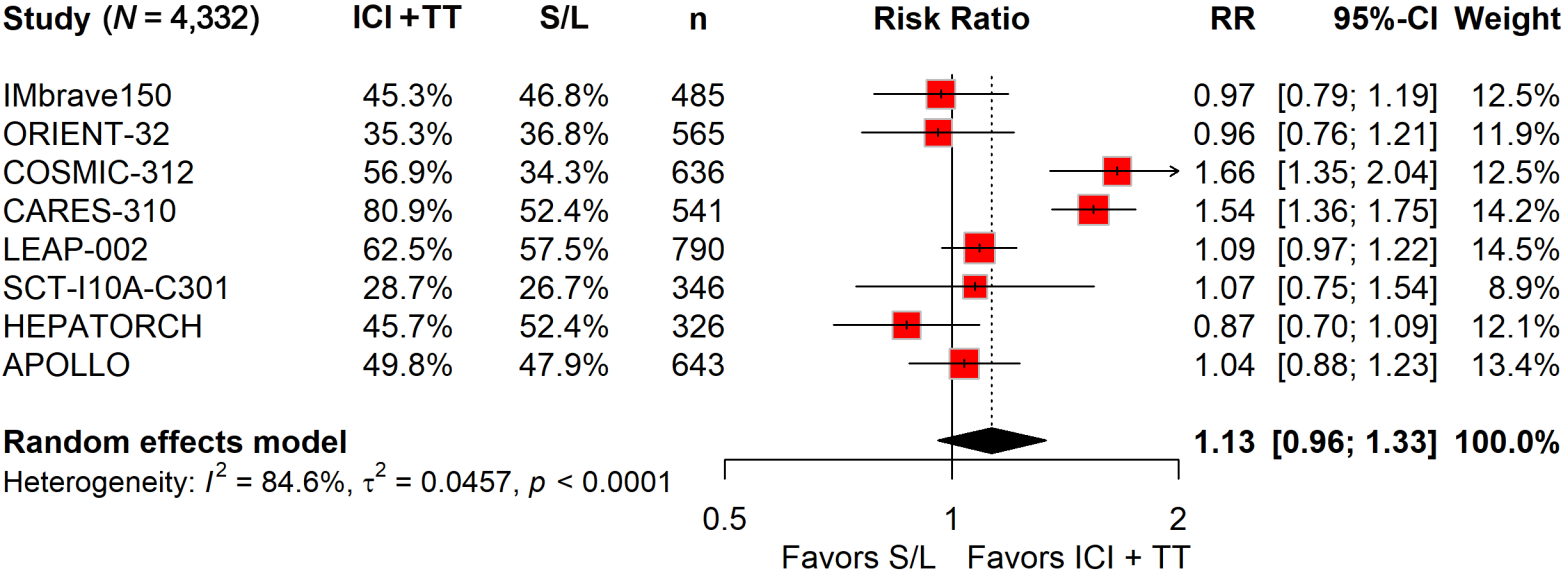
Forest plot of grade 3–5 treatment-related adverse events comparing immune checkpoint inhibitors plus targeted therapy *versus* sorafenib or lenvatinib monotherapy in patients with hepatocellular carcinoma.

**Figure 5:**
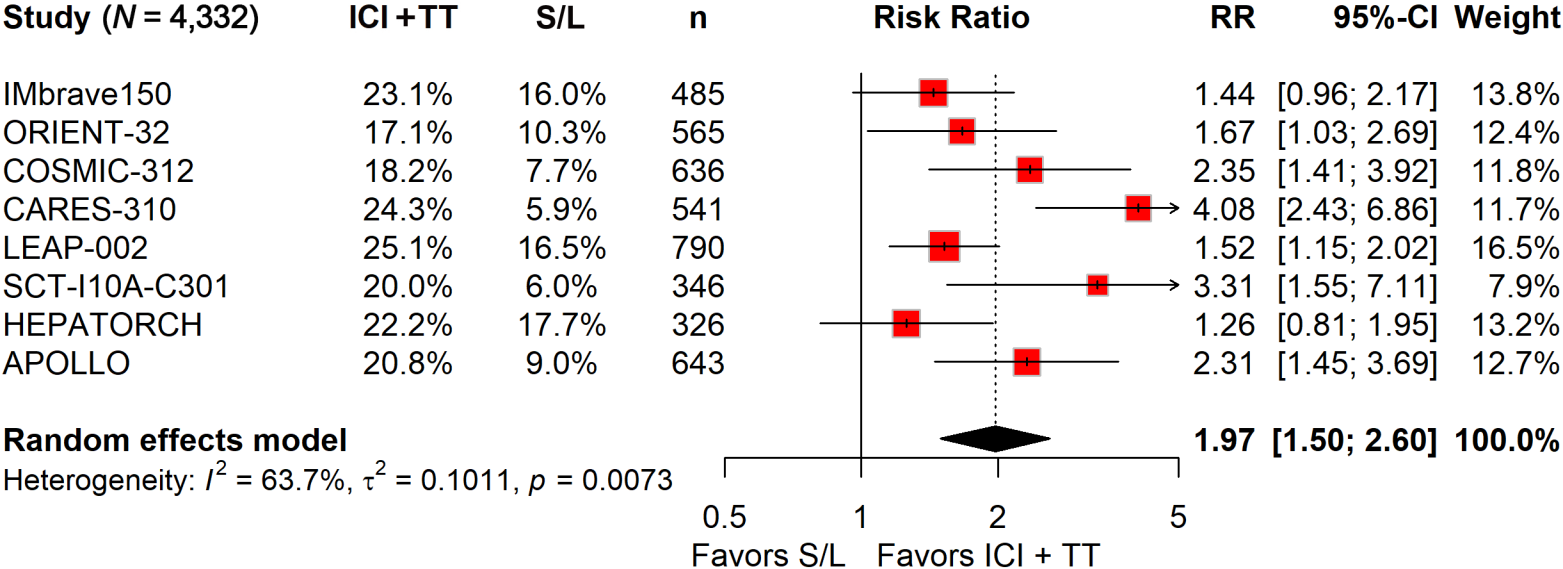
Forest plot of serious treatment-related adverse events comparing immune checkpoint inhibitors plus targeted therapy *versus* sorafenib or lenvatinib monotherapy in patients with hepatocellular carcinoma.

Among the 2,629 patients who received ICI in combination with TT, 1,349 (51.3%) experienced grade 3–5 TRAEs, compared to 798 of 1,703 patients (46.9%) treated with S/L monotherapy. The pooled RR indicated that the ICI-TT combination did not significantly increase the risk of grade 3–5 AEs (RR, 1.13; 95% CI, 0.96–1.33; **Figure 4**). Safety data on serious TRAEs were available from all eight trials. The incidence of serious TRAEs was 21.1% (556/2,629) in the ICI-TT group *versus* 11.5% (196/1,703) in the S/L monotherapy group. Pooled analysis revealed a significantly higher risk of serious TRAEs in patients receiving ICI-TT compared to those on S/L monotherapy (RR, 1.97; 95% CI, 1.50–2.60; **Figure 5**). In summary, while the risk of grade 3–5 TRAEs was comparable between groups, combination therapy was associated with a significantly higher incidence of serious TRAEs. This underscores the necessity of vigilant monitoring and proactive management when administering ICI-TT regimens, particularly in patients with borderline organ function.

### Subgroup analyses suggest heterogeneous benefits across sex, etiology, and disease burden

Given the limited power of individual studies to evaluate diverse clinically relevant subgroups, we conducted a series of subgroup analyses to better characterize the efficacy of ICI combined with TT in specific patient populations and to inform individualized precision treatment. The specific details of these subgroup analyses, including efficacy outcomes in different patient populations, are presented in **Table 2**.

**Table 2:** Subgroup analysis of progression-free survival and overall survival.

Subgroups	PFS		OS	
	Included studies	HR (95% CI)	Included studies	HR (95% CI)
Age				
<65 years	5	0.63 (0.52-0.76)	6	0.80 (0.66-0.97)
≥65 years	6	0.61 (0.48-0.76)	7	0.74 (0.62-0.89)
Sex				
Male	6	0.60 (0.53-0.67)	7	0.74 (0.64-0.86)
Female	6	0.69 (0.50-0.96)	7	0.83 (0.63-1.10)
ECOG PS				
0	7	0.63 (0.55-0.72)	8	0.79 (0.66-0.93)
1	7	0.59 (0.49-0.70)	8	0.67 (0.58-0.76)
AFP				
<400 ng/mL	7	0.60 (0.51-0.72)	8	0.71 (0.58-0.87)
≥400 ng/mL	7	0.57 (0.44-0.72)	8	0.61 (0.48-0.76)
BCLC stage				
B	7	0.63 (0.52-0.76)	8	0.63 (0.52-0.76)
C	7	0.61 (0.48-0.76)	8	0.70 (0.60-0.81)
Etiology				
HBC	7	0.55 (0.49-0.61)	8	0.66 (0.59-0.74)
HCV	4	0.63 (0.42-0.95)	5	0.73 (0.48-1.11)
Non-viral	4	0.81 (0.60-1.11)	5	1.00 (0.77-1.31)
MVI status				
No	7	0.64 (0.56-0.74)	8	0.78 (0.66-0.91)
Yes	7	0.53 (0.44-0.64)	8	0.65 (0.56-0.76)
EHS status				
No	7	0.65 (0.55-0.78)	8	0.79 (0.64-0.97)
Yes	7	0.58 (0.51-0.66)	8	0.71 (0.61-0.82)
MVI/EHS status				
No	7	0.72 (0.57-0.90)	8	0.77 (0.57-1.05)
Yes	7	0.57 (0.51-0.64)	8	0.70 (0.63-0.79)
Prior local therapy				
No	4	0.59 (0.48-0.73)	4	0.65 (0.54-0.78)
Yes	4	0.59 (0.49-0.71)	4	0.66 (0.49-0.90)

PFS, progression-free survival; OS, overall survival; HR, hazard ratio; CI, confidence interval; AFP, α-fetoprotein; BCLC, Barcelona Clinic Liver Cancer; HBV, Hepatitis B virus; HCV, Hepatitis C virus; ECOG PS, Eastern Cooperative Oncology Group performance status; MVI, macrovascular invasion; EHS extrahepatic spread.

Age-stratified subgroup analysis showed that ICI plus TT significantly prolonged PFS and OS in both patients younger than 65 years (PFS: HR, 0.63, 95% CI, 0.52–0.76; OS: HR, 0.80, 95% CI, 0.66–0.97) and those aged ≥65 years (PFS: HR, 0.61, 95% CI, 0.48–0.76; OS: HR, 0.74, 95% CI, 0.62–0.89) (**Supplementary Figure 2**).

Stratification by sex revealed that male patients derived significant benefits in both PFS (HR, 0.60; 95% CI, 0.53–0.67) and OS (HR, 0.74; 95% CI, 0.64–0.86) from ICI plus TT. For female patients, only PFS (HR, 0.69; 95% CI, 0.50–0.96) improvement was observed (**Supplementary Figure 3**).

Analysis by Eastern Cooperative Oncology Group (ECOG) status showed consistent efficacy regardless of performance status. For PFS: ECOG 0 (HR 0.63, 95% CI 0.55–0.72) and ECOG 1 (HR 0.59, 95% CI 0.49–0.70); for OS: ECOG 0 (HR 0.79, 95% CI 0.66–0.93) and ECOG 1 (HR 0.67, 95% CI 0.58–0.76) (**Supplementary Figure 4**).

Stratification by α-fetoprotein (AFP) level indicated significant PFS (AFP <400 ng/mL: HR 0.60, 95% CI 0.51–0.72; AFP ≥400 ng/mL: HR 0.57, 95% CI 0.44–0.72) and OS (AFP <400 ng/mL: HR 0.71, 95% CI 0.58–0.87; AFP ≥400 ng/mL: HR 0.70, 95% CI 0.60–0.81) benefits across all AFP levels (**Supplementary Figure 5**).

Patients with Barcelona Clinic Liver Cancer (BCLC) stage B showed significant PFS benefit from ICI plus TT (HR, 0.70; 95% CI, 0.54–0.90), whereas no significant OS improvement was detected. For stage C, significant improvements were observed in both PFS (HR, 0.58; 95% CI, 0.51–0.65) and OS (HR, 0.72; 95% CI, 0.64–0.81) (**Supplementary Figure 6**).

Among patients with HBV-related HCC, ICI plus TT significantly prolonged both PFS (HR, 0.55; 95% CI, 0.49–0.61) and OS (HR, 0.66; 95% CI, 0.59–0.74). For HCV-related cases, only PFS (HR, 0.63; 95% CI, 0.42–0.95) was significantly improved, with no OS benefit observed. Patients with non-viral etiology showed no significant improvements in either endpoint (**Supplementary Figure 7**).

Macrovascular invasion (MVI) status did not affect efficacy, with significant PFS (no MVI: HR, 0.64; 95% CI, 0.56–0.74; MVI: HR, 0.53; 95% CI 0.44–0.64) and OS (no MVI: HR, 0.78; 95% CI, 0.66–0.91; MVI: HR, 0.65; 95% CI, 0.56–0.76) benefits observed regardless of MVI presence (**Supplementary Figure 8**).

Subgroup analysis of extrahepatic spread (EHS) confirmed that ICI plus TT significantly improves both PFS and OS regardless of EHS status: for PFS, benefits were observed in patients without EHS (HR, 0.65, 95% CI, 0.55–0.78) and those with EHS (HR, 0.58, 95% CI, 0.51–0.66); for OS, significant benefits were also seen in patients without EHS (HR, 0.79, 95% CI, 0.64–0.97) and with EHS (HR, 0.71, 95% CI, 0.62–0.82) (**Supplementary Figure 9**).

Combined MVI/EHS status analysis revealed significant PFS benefit in patients without these features (HR, 0.72; 95% CI; 0.57–0.90) and with either/both features (HR, 0.57; 95% CI, 0.51–0.64). OS benefit was significant only in patients with either/both features (HR, 0.70; 95% CI, 0.63–0.79) (**Supplementary Figure 10**).

Prior local therapy history did not influence outcomes, with significant PFS (no prior: HR, 0.59; 95% CI, 0.48–0.73; prior: HR, 0.59; 95% CI, 0.49–0.71) and OS (no prior: HR, 0.65; 95% CI, 0.54–0.78; prior: HR, 0.66; 95% CI, 0.49–0.90) benefits observed irrespective of treatment history (**Supplementary Figure 11**).

Taken together, subgroup findings suggest potential heterogeneity of benefit by etiology, sex, and tumor burden. However, these analyses were exploratory and underpowered, and therefore should be interpreted as hypothesis-generating rather than definitive.

### Risk of bias and sensitivity analysis

The risk of bias across the included trials is graphically summarized in **Supplementary Figures 12, Supplementary Figures 13**. All eight studies were randomized, open-label trials with investigator-assessed outcomes and pre-specified analysis strategies. As a result, all studies were deemed to have a low risk of selection, detection, attrition, and reporting bias, but a high risk of performance bias due to the open-label design. Funnel plots and Egger’s tests revealed no evidence of publication bias (**Supplementary Figure 14**). Sensitivity analyses confirmed the robustness of the pooled results (**Supplementary Figure 15**). Overall, the included studies demonstrated a low risk of bias in key domains, and the primary findings were robust upon sensitivity analysis. The main limitation, consistent across all trials, was the high risk of performance bias inherent to the open-label design.

## Discussion

This comprehensive meta-analysis of eight phase 3 RCTs, encompassing 4,379 patients with u/aHCC, provides robust evidence supporting the superiority of first-line ICI plus TT combinations over S/L monotherapy. The key findings demonstrate significant improvements in ORR (OR 3.93), PFS (HR 0.62), and OS (HR 0.71) with combination therapy. While the risk of grade 3–5 TRAEs was not significantly elevated, the 97% increase in serious TRAEs (RR 1.97) underscores the need for careful patient selection and vigilant monitoring. These findings consolidate the evolving role of ICI-based regimens as the cornerstone of systemic therapy in u/aHCC and highlight the need for thoughtful patient selection to balance efficacy with tolerability.

Our findings are broadly consistent with previous meta-analyses that established the superiority of ICI-TT combinations over sorafenib or lenvatinib. Nonetheless, our study provides incremental value in three key aspects. First, it incorporates the most comprehensive dataset to date, pooling eight phase 3 RCTs with 4,379 patients, whereas earlier reviews included only four to six trials. Second, it integrates the latest evidence from recently reported global and regional phase 3 trials (HEPATORCH, APOLLO, SCT-I10A-C301), which were not available in prior analyses and substantially expand the evidence base. Third, it offers the most detailed subgroup evaluation to date, spanning etiology, sex, AFP levels, ECOG status, BCLC stage, and prior therapy. By critically situating our work within the existing literature, we highlight how our analysis extends beyond confirmatory findings to generate clinically relevant, hypothesis-generating insights.

Compared with prior studies, Li et al. synthesized four phase 3 RCTs and confirmed that PD-1/PD-L1 inhibitors plus antiangiogenic agents improved OS and PFS versus sorafenib, but their work was limited by smaller sample size and fewer subgroup data (20). Zhou et al. performed a network meta-analysis comparing anti-PD-1/L1 plus VEGF antibody with anti-PD-1/L1 plus VEGFR-TKI, but they did not focus on pooled head-to-head evidence against sorafenib or lenvatinib (21). Fulgenzi et al. also compared novel first-line strategies, identifying atezolizumab plus bevacizumab as a benchmark, but their dataset was restricted to studies published before early 2022 (18). Zhu et al. included five RCTs and confirmed improved OS and PFS with PD-1/PD-L1 plus anti-angiogenic therapy, although their analysis lacked the most recently completed phase 3 trials (23). Li et al. conducted a large-scale network meta-analysis involving 17 trials, but their scope extended beyond immunotherapy-based regimens and incorporated HAIC-FO and TACE-based strategies, limiting the specificity of conclusions for ICI-TT (19). She et al. addressed a similar question, but their work did not integrate the newest phase 3 evidence (22).

The observed 38% reduction in progression risk and 29% reduction in mortality risk establish ICI-TT regimens as the new therapeutic benchmark, surpassing historical outcomes achieved with tyrosine kinase inhibitor (TKI) monotherapy. The near-quadrupling of objective response rates holds particular clinical relevance for patients requiring rapid symptom control or tumor downstaging. Importantly, the absence of a significant increase in grade 3–5 TRAEs suggests manageable toxicity profiles in experienced centers. However, the substantially elevated risk of serious TRAEs necessitates rigorous patient assessment, especially for those with compromised liver function (Child-Pugh B), significant portal hypertension, or autoimmune comorbidities. This safety-efficacy balance forms the cornerstone of clinical decision-making when considering combination therapy.

The biological rationale for successful ICI-TT synergy centers on reversing the characteristically immunosuppressive tumor microenvironment of HCC (27, 28). VEGF blockade normalizes the aberrant tumor vasculature, enhancing T-cell infiltration while simultaneously reducing immunosuppressive regulatory T cells and myeloid-derived suppressor cells (29). PD-1/PD-L1 inhibition then reinvigorates exhausted CD8+ T-cells and enhances antigen presentation (30).

Subgroup analyses revealed heterogeneous responses across patient populations, offering critical guidance for personalized treatment. PFS benefits were consistent across age groups, with significant improvements observed in both patients <65 years (HR 0.63) and those ≥65 years (HR 0.61). For OS, significant benefits were also demonstrated in both younger (HR 0.80) and older (HR 0.74) patients, supporting the efficacy of combination therapy across the adult age spectrum, provided liver function and performance status are adequate. Male patients derived significant benefits in both PFS (HR 0.60) and OS (HR 0.74). In contrast, female patients showed a significant improvement in PFS (HR 0.69) but not in OS. These findings warrant cautious interpretation given the smaller female sample size and possible sex-related immunological differences, underscoring the need for further exploration of sex-specific tumor-immune dynamics. Consistent efficacy across ECOG 0 and 1 patients reinforces the generalizability of ICI-TT benefits across a broad spectrum of functional status. Furthermore, patients with high AFP (≥400 ng/mL) demonstrated significant PFS and OS benefits comparable to those with low AFP, challenging the notion that AFP is a negative prognostic marker for immunotherapy (31).

Etiology-specific analyses yielded clinically relevant patterns. Patients with HBV-related HCC experienced robust improvements in both PFS (HR 0.55) and OS (HR 0.66), solidifying ICI-TT as the preferred first-line approach for this population. For HCV-related HCC, a significant PFS benefit (HR 0.63) was observed, but this did not translate into a significant OS improvement. Most notably, patients with non-viral HCC (predominantly NAFLD/NASH etiology) showed no significant improvements in either PFS or OS. This suggests distinct tumor biology in metabolic syndrome-driven hepatocarcinogenesis, characterized by profoundly immunosuppressive features including CD8^+^ T-cell exhaustion, altered gut microbiota, and fibrotic stroma (32, 33).

Tumor burden and aggressiveness features further refined benefit predictions. Patients with BCLC stage B disease derived a significant PFS benefit (HR 0.70) but not a significant OS improvement, possibly due to effective crossover to second-line therapies after progression. Conversely, those with stage C disease demonstrated significant improvements in both PFS (HR 0.58) and OS (HR 0.72), highlighting the particular value of combination therapy in biologically aggressive disease. The presence of MVI or EHS were significant determinants of therapeutic benefit. Significant OS improvement was confined to patients with MVI (HR 0.65) and/or EHS (HR 0.71)—populations with historically dismal prognoses—supporting early, aggressive combination therapy in these high-risk subgroups. The analysis of prior local therapy revealed consistent PFS and OS benefits regardless of treatment history, suggesting ICI-TT combinations can overcome resistance to prior interventions, potentially by reversing therapy-induced immunosuppression.

Our findings have significant clinical implications. First, they reinforce ICI-TT combinations as the preferred first-line treatment option for eligible patients with u/aHCC, aligning with recent guideline updates. Second, the identification of subgroups with differential benefit supports a more nuanced, precision medicine approach, emphasizing the need for biomarkers predictive of response and toxicity. Third, the elevated risk of serious AEs calls for the development of standardized management protocols and patient education to optimize safety.

Despite the strengths of this meta-analysis, including the large pooled sample size and rigorous methodology, several limitations merit discussion. First, the heterogeneity among included regimens–encompassing diverse combinations of PD-1/PD-L1 inhibitors with VEGF monoclonal antibodies or TKIs–inherently assumes class effects that may not uniformly hold across specific agents. Furthermore, all included trials employed open-label designs, introducing potential performance and reporting bias despite pre-specified outcome assessment protocols. Moreover, subgroup analyses, while informative, were statistically underpowered for smaller populations such as non-viral HCC patients or females Additionally, variable follow-up durations across trials, with mature OS data unavailable for newer studies like APOLLO and HEPATORCH, may affect long-term outcome assessments. Finally, systematic exclusion of Child-Pugh B patients from most of the included trials limits generalizability to this clinically important population with compromised hepatic function.

Future research directions should address these knowledge gaps while building upon current successes. Biomarker development remains paramount, as PD-L1 expression demonstrates inconsistent predictive value in HCC. Emerging candidates including tumor mutational burden, inflammatory gene signatures, and gut microbiome profiles require rigorous validation. Early on-treatment biomarkers such as AFP response (defined as ≥20% decline at week 6) show promise as early efficacy indicators (34, 35). Novel agents warrant exploration, particularly bispecific antibodies (e.g., anti-PD-1/VEGF inhibitors). For the challenging non-viral HCC subgroup, TGF-β inhibitors merit investigation to counteract the profound stromal immunosuppression characteristic of these tumors. Sequencing strategies post-progression represents another critical knowledge gap, requiring prospective evaluation of optimal therapeutic approaches after ICI-TT failure. Finally, given the substantial costs of combination immunotherapies, rigorous cost-effectiveness analyses are essential to ensure global accessibility and sustainability of these advances.

## Conclusion

This meta-analysis solidifies ICI-TT combinations as the cornerstone of first-line therapy for u/aHCC, demonstrating unequivocal superiority over S/L monotherapy across survival and response endpoints. The consistent benefit observed across most clinically relevant subgroups–particularly those with high-risk features including MVI, EHS, and HBV etiology–supports broad applicability in routine practice. However, the significantly increased risk of serious TRAEs necessitates stringent patient selection criteria. Future efforts must prioritize biomarker-driven personalization, novel combination strategies for non-viral HCC subtypes, and interventions to enhance safety without compromising efficacy. As the therapeutic landscape continues to evolve, this comprehensive analysis provides a robust foundation for evidence-based clinical decision-making while underscoring the transformative impact of immunotherapy integration in HCC management.

## Data Availability

The original contributions presented in the study are included in the article/**Supplementary Material**. Further inquiries can be directed to the corresponding author.
