# Supplementary material for: First-line immune checkpoint inhibitors plus targeted therapy versus sorafenib or lenvatinib monotherapy for unresectable or advanced hepatocellular carcinoma: a meta-analysis of phase 3 trials
Source: Front Immunol. 2025 Oct 15;16:1667793. doi: 10.3389/fimmu.2025.1667793 (PMC12568680; doi:10.3389/fimmu.2025.1667793)
Supplement: Supplementary file 1 [file DataSheet1.docx]

**Supplementary Material**

**First-line immune checkpoint inhibitors plus targeted therapy versus sorafenib or lenvatinib monotherapy for unresectable or advanced hepatocellular carcinoma: a meta-analysis of phase 3 trials**

**Supplementary Table 1.** Details of search strategy.

**Supplementary Figure 1.** PRISMA flow diagram of study selection.

**Supplementary Figure 2.** Forest plot of progression-free survival (**A**) and overall survival (**B**) comparing immune checkpoint inhibitors plus targeted therapy *versus* sorafenib or lenvatinib monotherapy in patients with hepatocellular carcinoma stratified by age.

**Supplementary Figure 3.** Forest plot of progression-free survival (**A**) and overall survival (**B**) comparing immune checkpoint inhibitors plus targeted therapy *versus* sorafenib or lenvatinib monotherapy in patients with hepatocellular carcinoma stratified by sex.

**Supplementary Figure 4.** Forest plot of progression-free survival (**A**) and overall survival (**B**) comparing immune checkpoint inhibitors plus targeted therapy *versus* sorafenib or lenvatinib monotherapy in patients with hepatocellular carcinoma stratified by Eastern Cooperative Oncology Group performance status.

**Supplementary Figure 5.** Forest plot of progression-free survival (**A**) and overall survival (**B**) comparing immune checkpoint inhibitors plus targeted therapy *versus* sorafenib or lenvatinib monotherapy in patients with hepatocellular carcinoma stratified by α-fetoprotein level.

**Supplementary Figure 6.** Forest plot of progression-free survival (**A**) and overall survival (**B**) comparing immune checkpoint inhibitors plus targeted therapy *versus* sorafenib or lenvatinib monotherapy in patients with hepatocellular carcinoma stratified by Barcelona Clinic Liver Cancer stage.

**Supplementary Figure 7.** Forest plot of progression-free survival (**A**) and overall survival (**B**) comparing immune checkpoint inhibitors plus targeted therapy *versus* sorafenib or lenvatinib monotherapy in patients with hepatocellular carcinoma stratified by etiology.

**Supplementary Figure 8.** Forest plot of progression-free survival (**A**) and overall survival (**B**) comparing immune checkpoint inhibitors plus targeted therapy *versus* sorafenib or lenvatinib monotherapy in patients with hepatocellular carcinoma stratified by macrovascular invasion status.

**Supplementary Figure 9.** Forest plot of progression-free survival (**A**) and overall survival (**B**) comparing immune checkpoint inhibitors plus targeted therapy *versus* sorafenib or lenvatinib monotherapy in patients with hepatocellular carcinoma stratified by extrahepatic spread status.

**Supplementary Figure 10.** Forest plot of progression-free survival (**A**) and overall survival (**B**) comparing immune checkpoint inhibitors plus targeted therapy *versus* sorafenib or lenvatinib monotherapy in patients with hepatocellular carcinoma stratified by macrovascular invasion and/or extrahepatic spread status.

**Supplementary Figure 11.** Forest plot of progression-free survival (**A**) and overall survival (**B**) comparing immune checkpoint inhibitors plus targeted therapy *versus* sorafenib or lenvatinib monotherapy in patients with hepatocellular carcinoma stratified by history of prior local therapy (received *vs.* not received).

**Supplementary Figure 12.** Risk of bias graph.

**Supplementary Figure 13.** Risk of bias summary.

**Supplementary Figure 14.** Funnel plots and Egger’s tests for progression-free survival and overall survival.

**Supplementary Figure 15.** Sensitivity analyses for included studies on progression-free survival (**A**) and overall survival (**B**) examined by leaving-one-out approach.

**Supplementary Table 1.** Details of search strategy.

| **Database** | **Search strategy** |
| --- | --- |
| ***PubMed*** | (((Immunotherapy[Title/Abstract] OR "Immune Checkpoint Inhibitors"[Title/Abstract] OR Atezolizumab[Title/Abstract] OR Pembrolizumab[Title/Abstract] OR Sintilimab[Title/Abstract] OR Camrelizumab[Title/Abstract] OR Toripalimab[Title/Abstract] OR Penpulimab[Title/Abstract])) AND (("Targeted Therapy"[Title/Abstract] OR Bevacizumab[Title/Abstract] OR Sorafenib[Title/Abstract] OR Lenvatinib[Title/Abstract]))) AND (("Hepatocellular Carcinoma"[Title/Abstract] OR "Liver Cancer"[Title/Abstract])) |


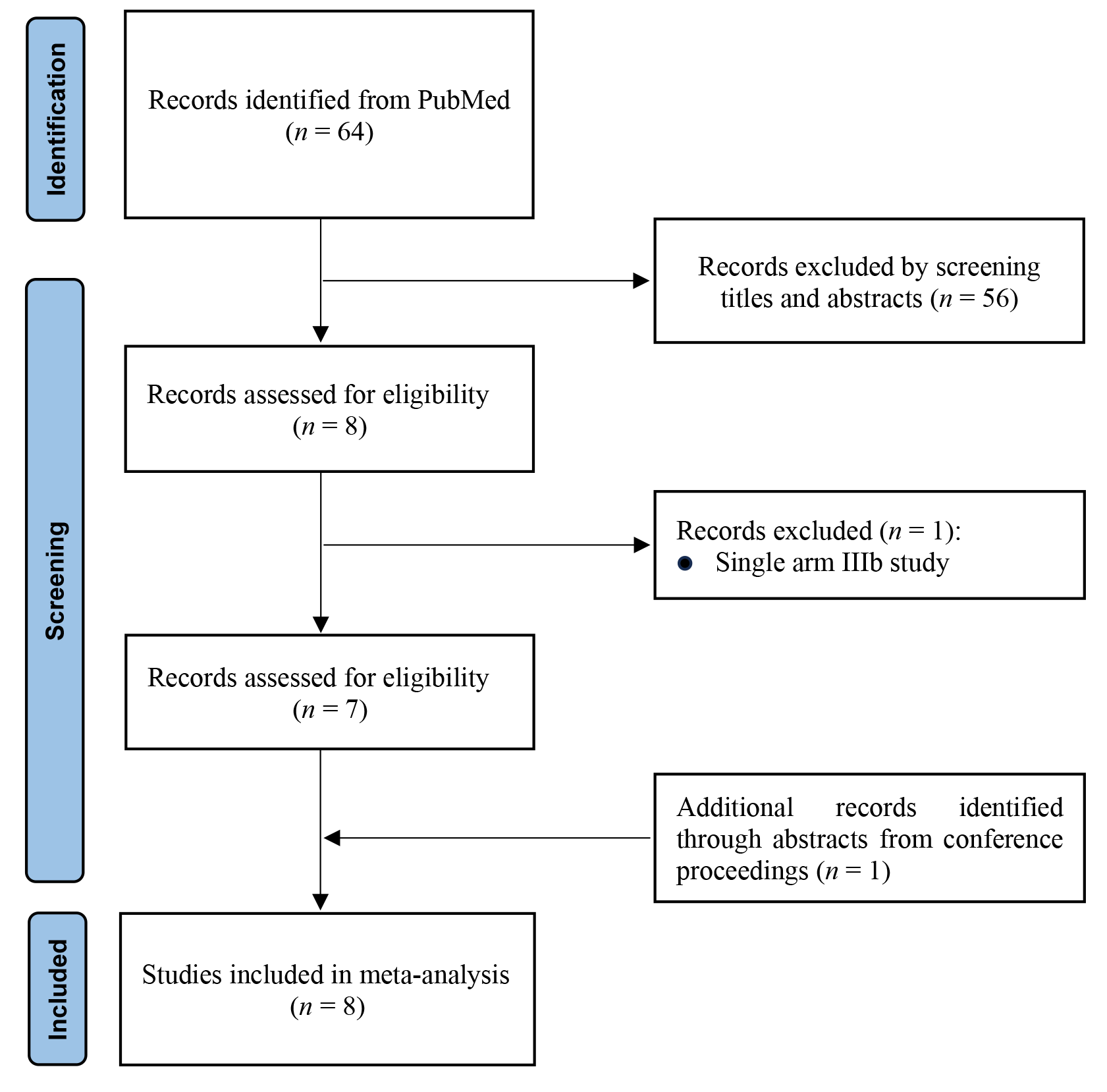


**Supplementary Figure 1.** PRISMA flow diagram of study selection.

**
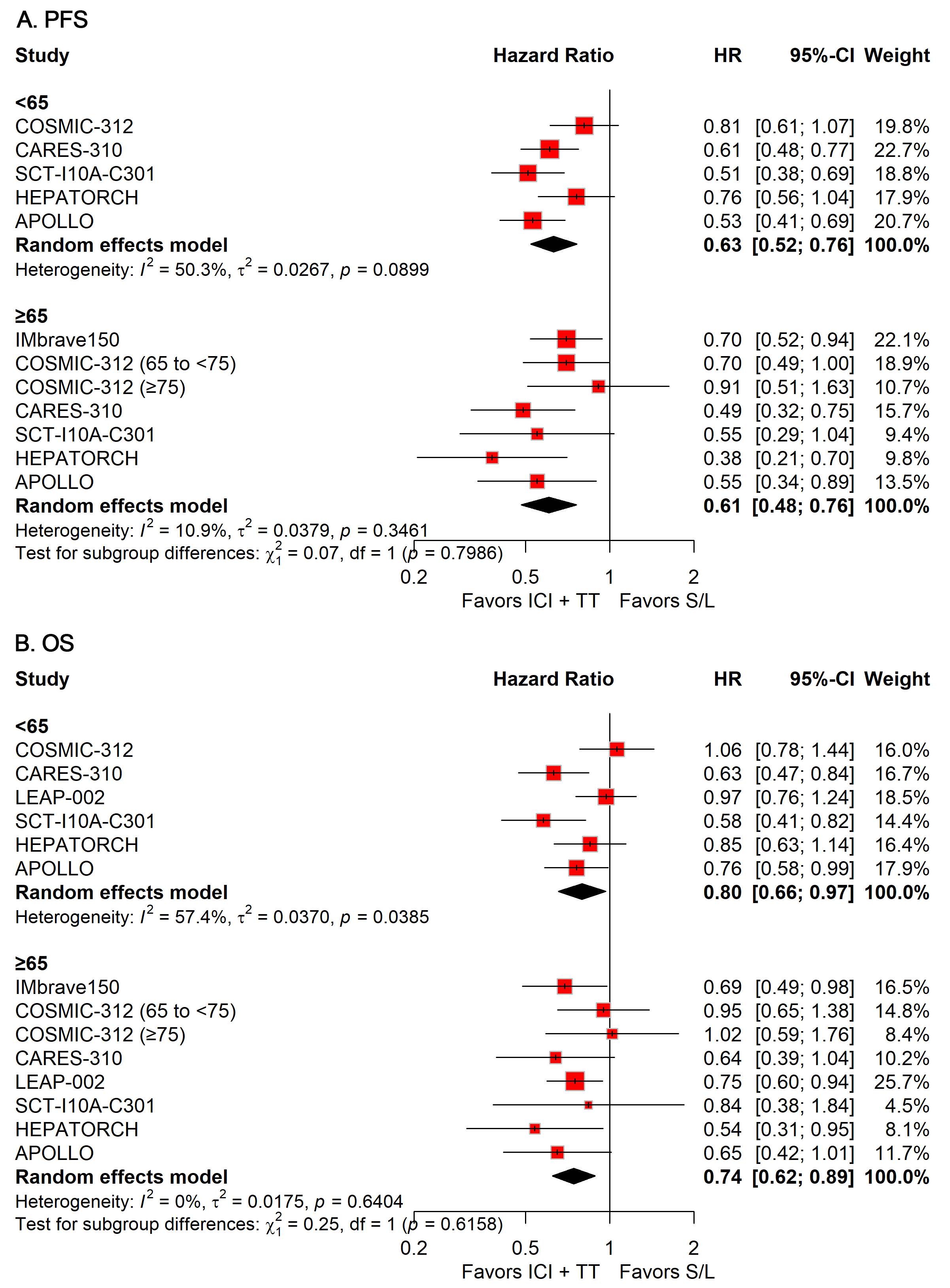
**

**Supplementary Figure 2.** Forest plot of progression-free survival (**A**) and overall survival (**B**) comparing immune checkpoint inhibitors plus targeted therapy *versus* sorafenib or lenvatinib monotherapy in patients with hepatocellular carcinoma stratified by age.

**
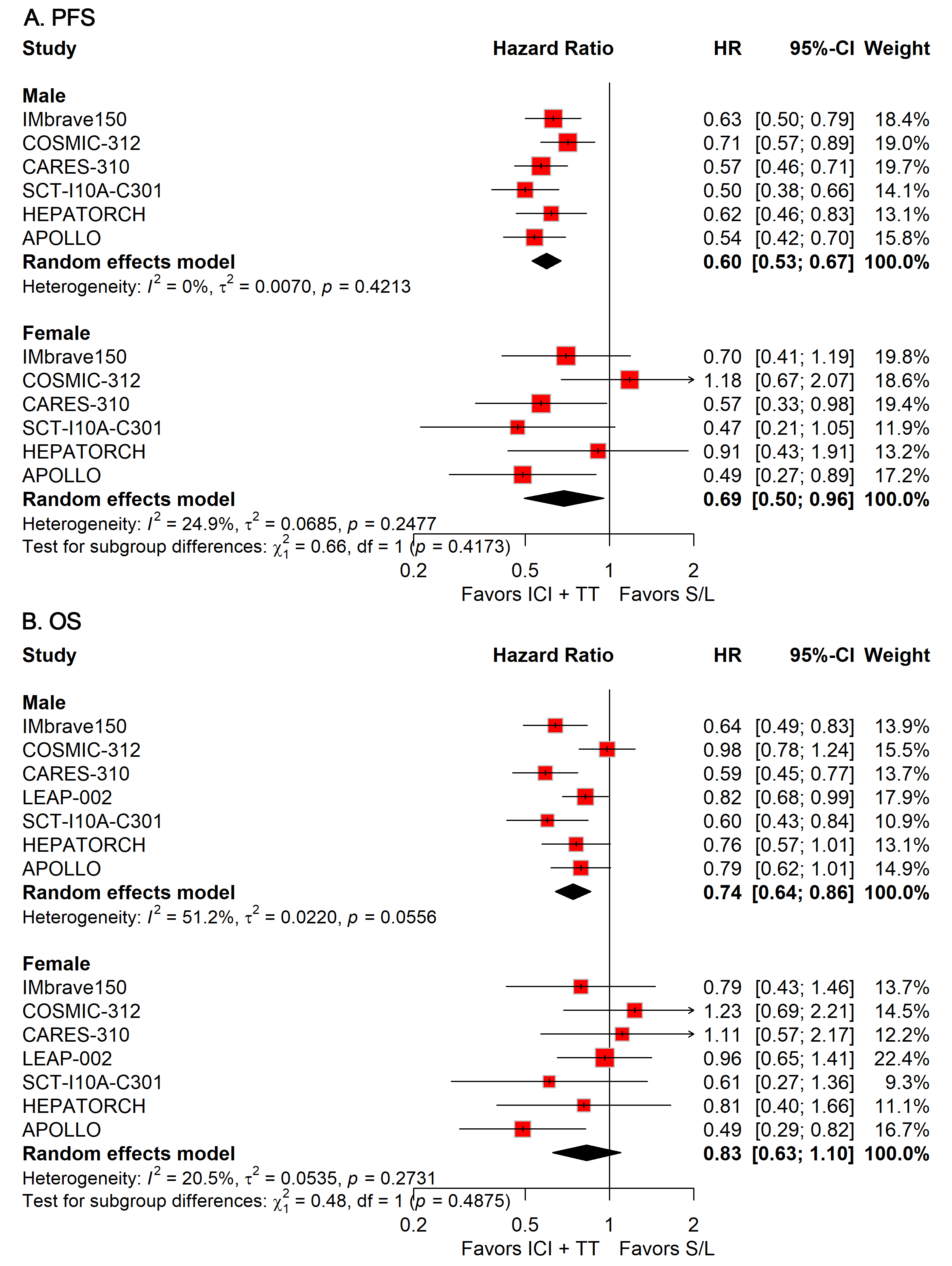
**

**Supplementary Figure 3.** Forest plot of progression-free survival (**A**) and overall survival (**B**) comparing immune checkpoint inhibitors plus targeted therapy *versus* sorafenib or lenvatinib monotherapy in patients with hepatocellular carcinoma stratified by sex.

**
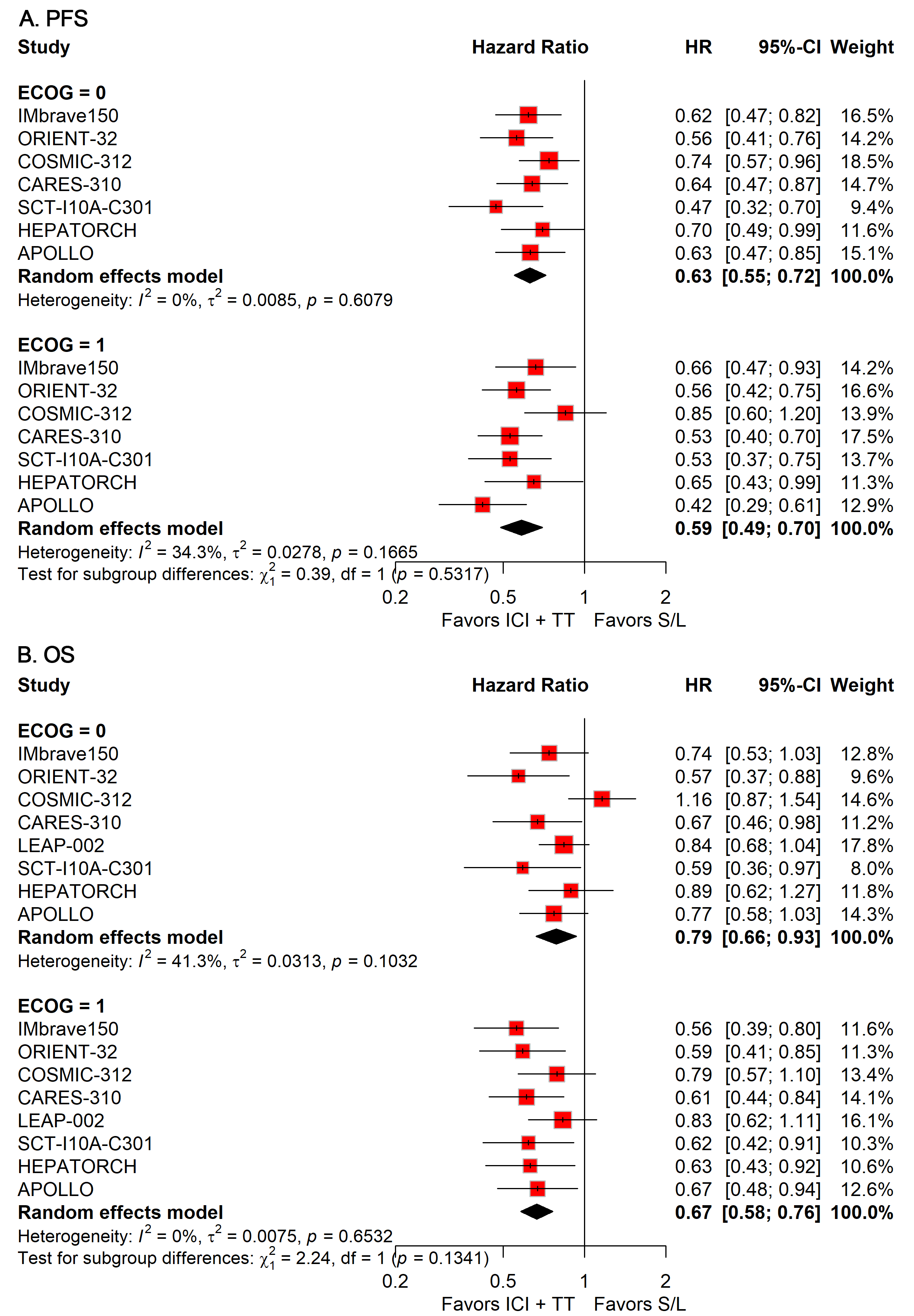
**

**Supplementary Figure 4.** Forest plot of progression-free survival (**A**) and overall survival (**B**) comparing immune checkpoint inhibitors plus targeted therapy *versus* sorafenib or lenvatinib monotherapy in patients with hepatocellular carcinoma stratified by Eastern Cooperative Oncology Group performance status.

**
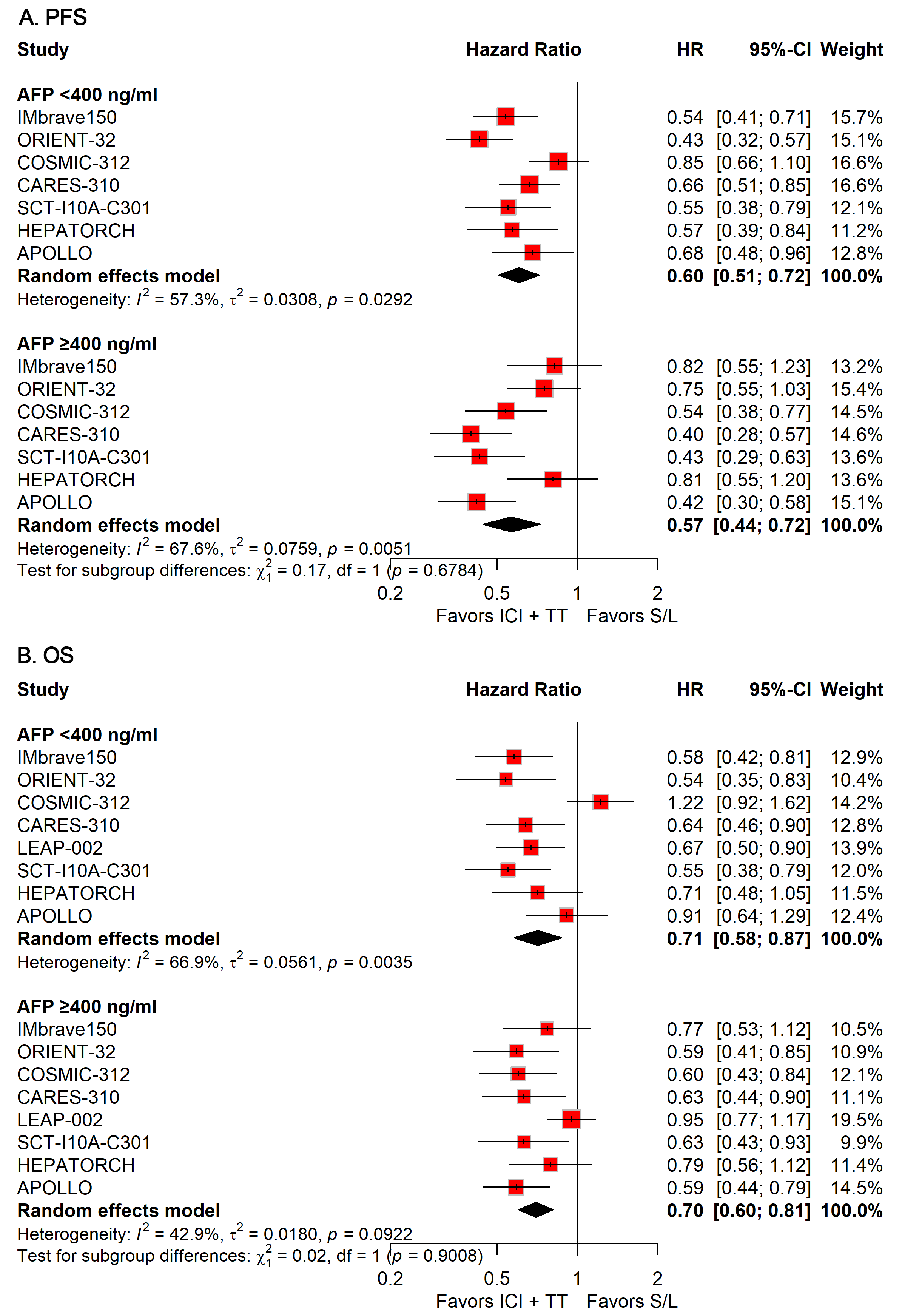
**

**Supplementary Figure 5.** Forest plot of progression-free survival (**A**) and overall survival (**B**) comparing immune checkpoint inhibitors plus targeted therapy *versus* sorafenib or lenvatinib monotherapy in patients with hepatocellular carcinoma stratified by α-fetoprotein level.

**
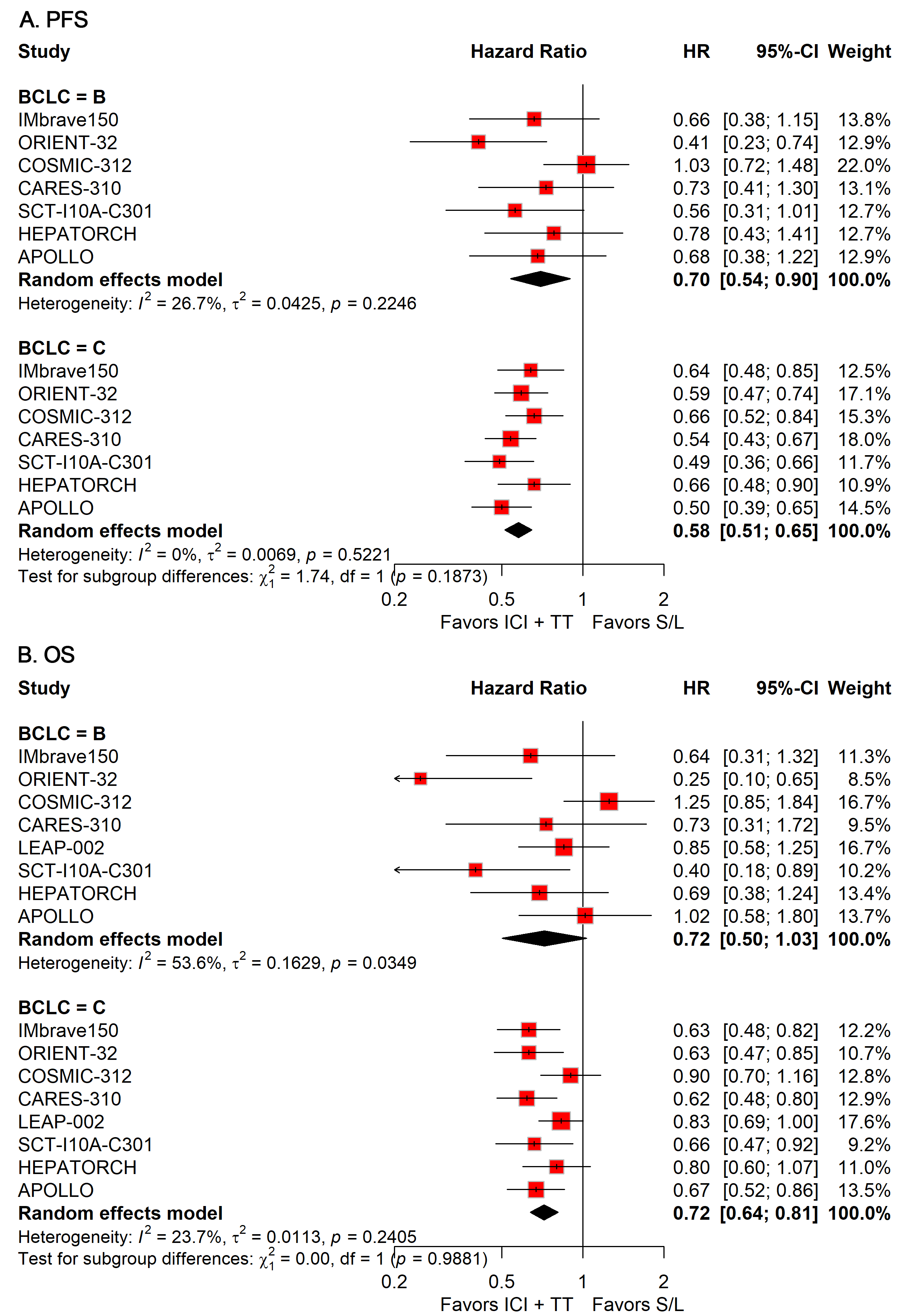
**

**Supplementary Figure 6.** Forest plot of progression-free survival (**A**) and overall survival (**B**) comparing immune checkpoint inhibitors plus targeted therapy *versus* sorafenib or lenvatinib monotherapy in patients with hepatocellular carcinoma stratified by Barcelona Clinic Liver Cancer stage.


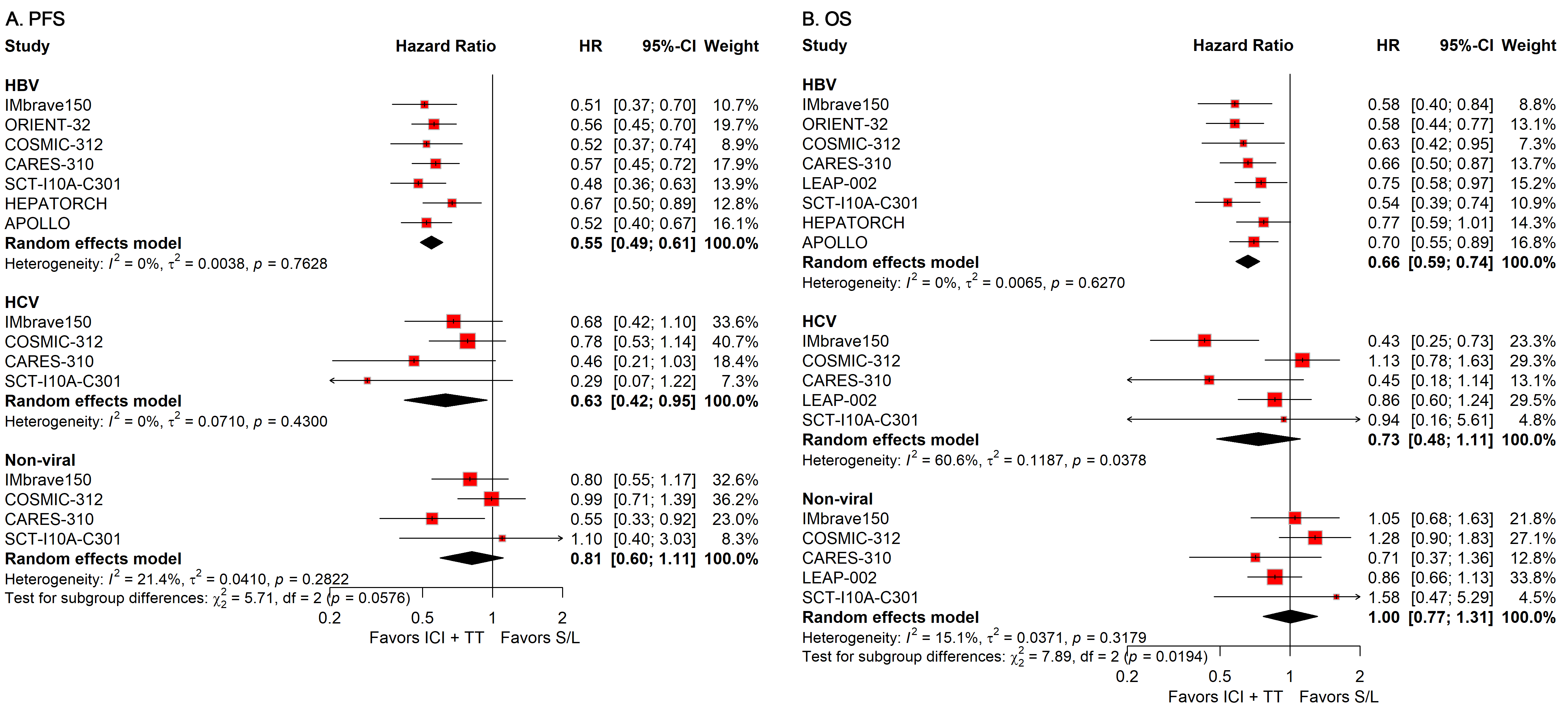


**Supplementary Figure 7.** Forest plot of progression-free survival (**A**) and overall survival (**B**) comparing immune checkpoint inhibitors plus targeted therapy *versus* sorafenib or lenvatinib monotherapy in patients with hepatocellular carcinoma stratified by etiology.


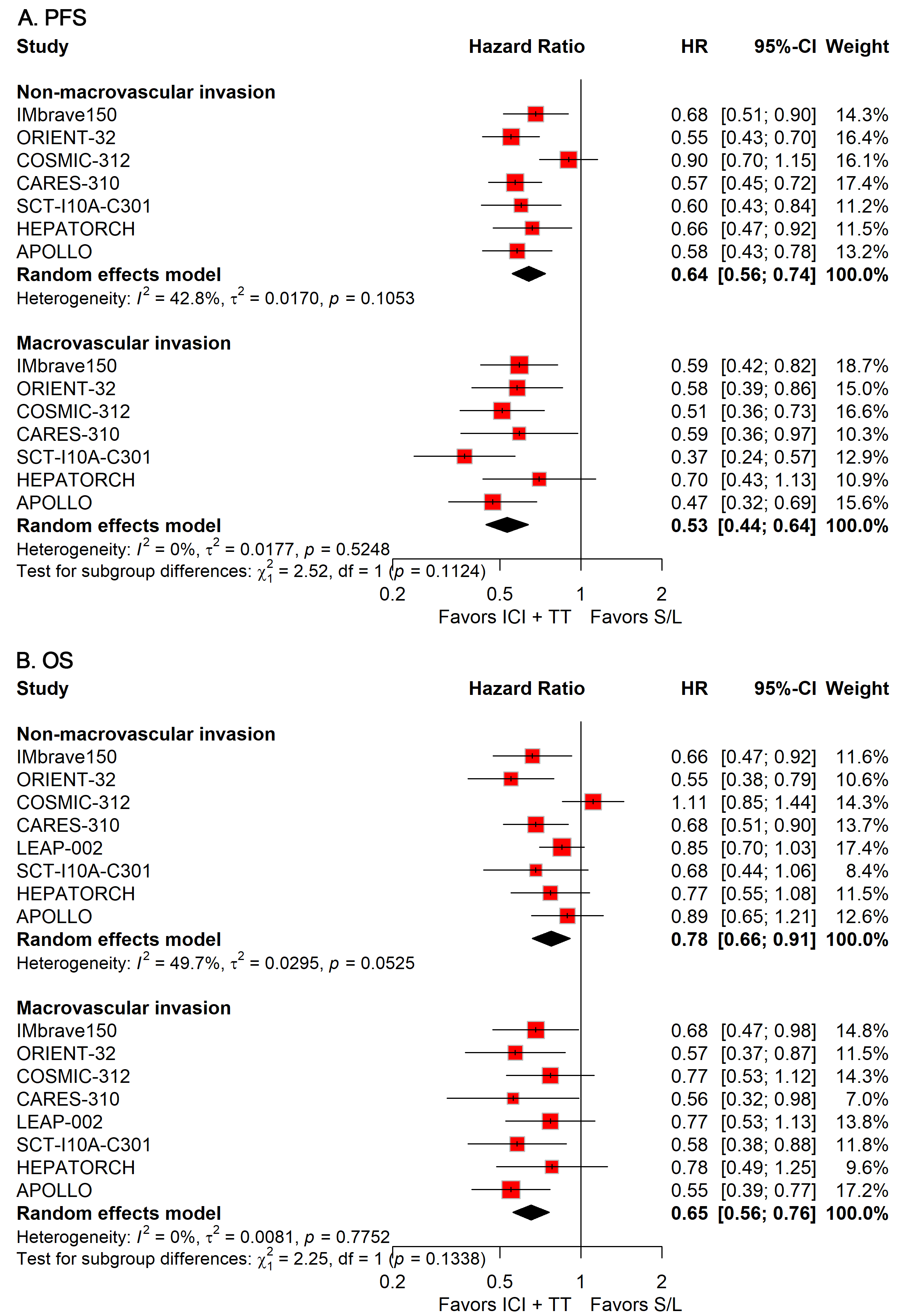


**Supplementary Figure 8.** Forest plot of progression-free survival (**A**) and overall survival (**B**) comparing immune checkpoint inhibitors plus targeted therapy *versus* sorafenib or lenvatinib monotherapy in patients with hepatocellular carcinoma stratified by macrovascular invasion status.


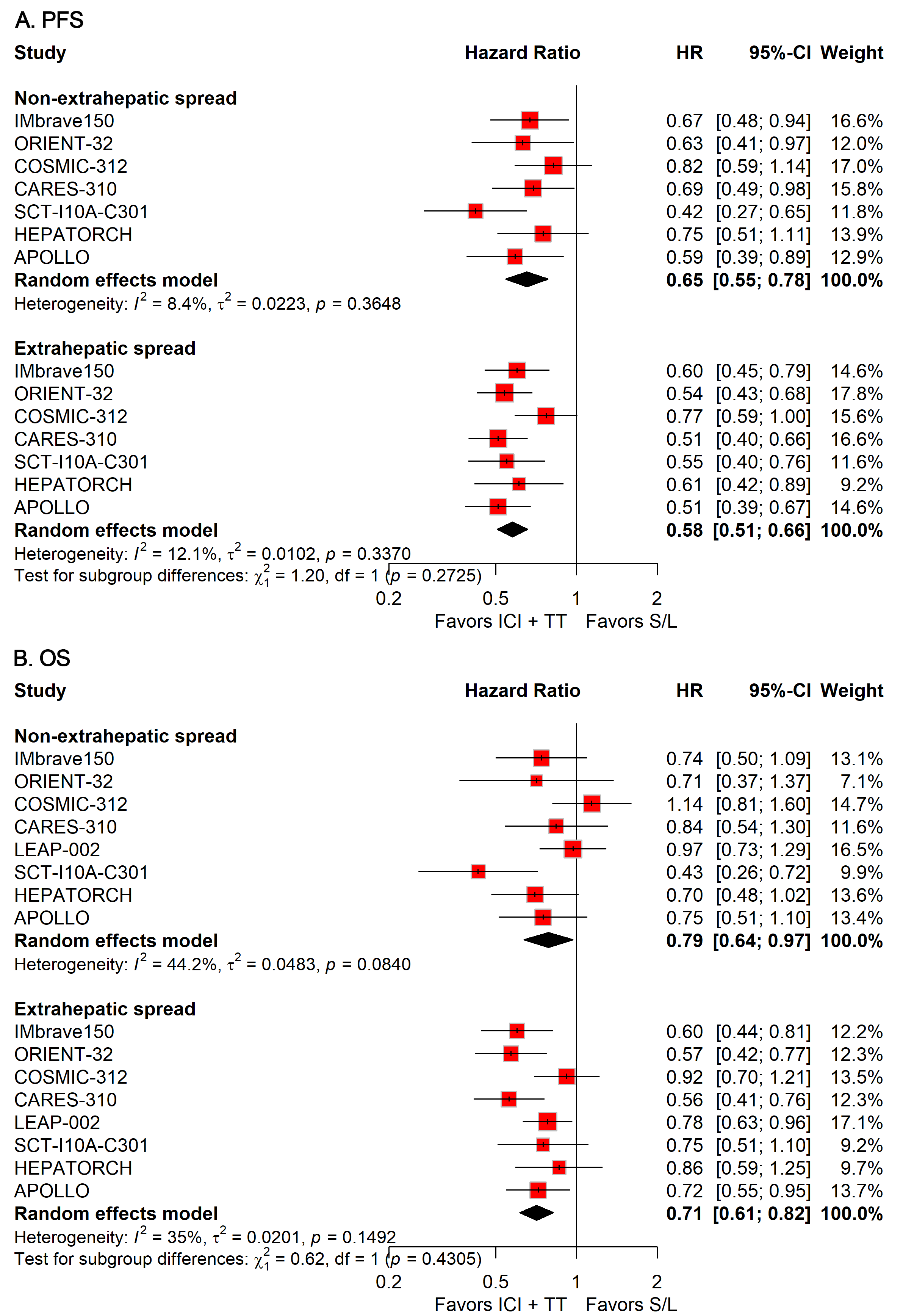


**Supplementary Figure 9.** Forest plot of progression-free survival (**A**) and overall survival (**B**) comparing immune checkpoint inhibitors plus targeted therapy *versus* sorafenib or lenvatinib monotherapy in patients with hepatocellular carcinoma stratified by extrahepatic spread status.


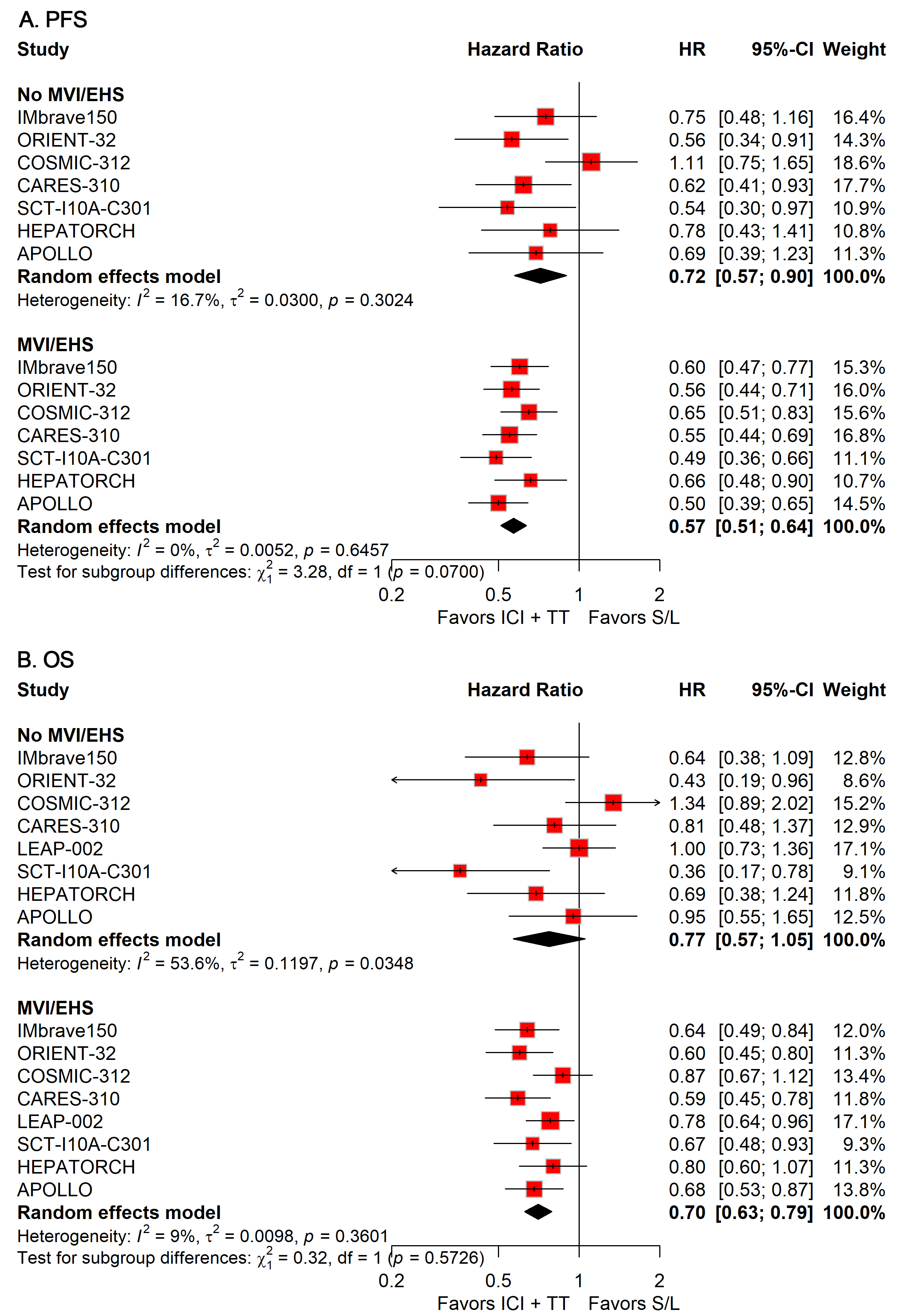


**Supplementary Figure 10.** Forest plot of progression-free survival (**A**) and overall survival (**B**) comparing immune checkpoint inhibitors plus targeted therapy *versus* sorafenib or lenvatinib monotherapy in patients with hepatocellular carcinoma stratified by macrovascular invasion and/or extrahepatic spread status.


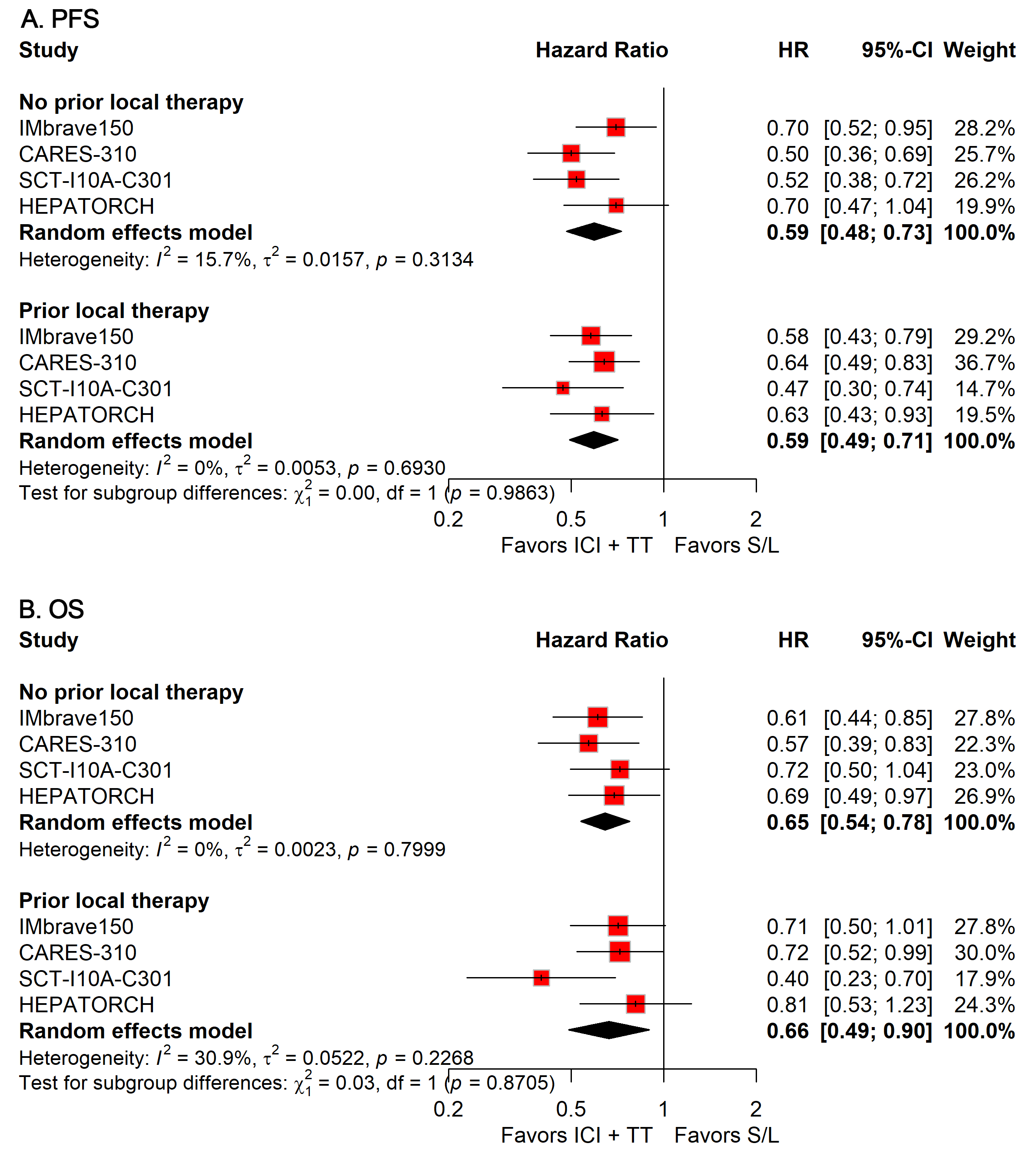


**Supplementary Figure 11.** Forest plot of progression-free survival (**A**) and overall survival (**B**) comparing immune checkpoint inhibitors plus targeted therapy *versus* sorafenib or lenvatinib monotherapy in patients with hepatocellular carcinoma stratified by history of prior local therapy (received *vs.* not received).

**
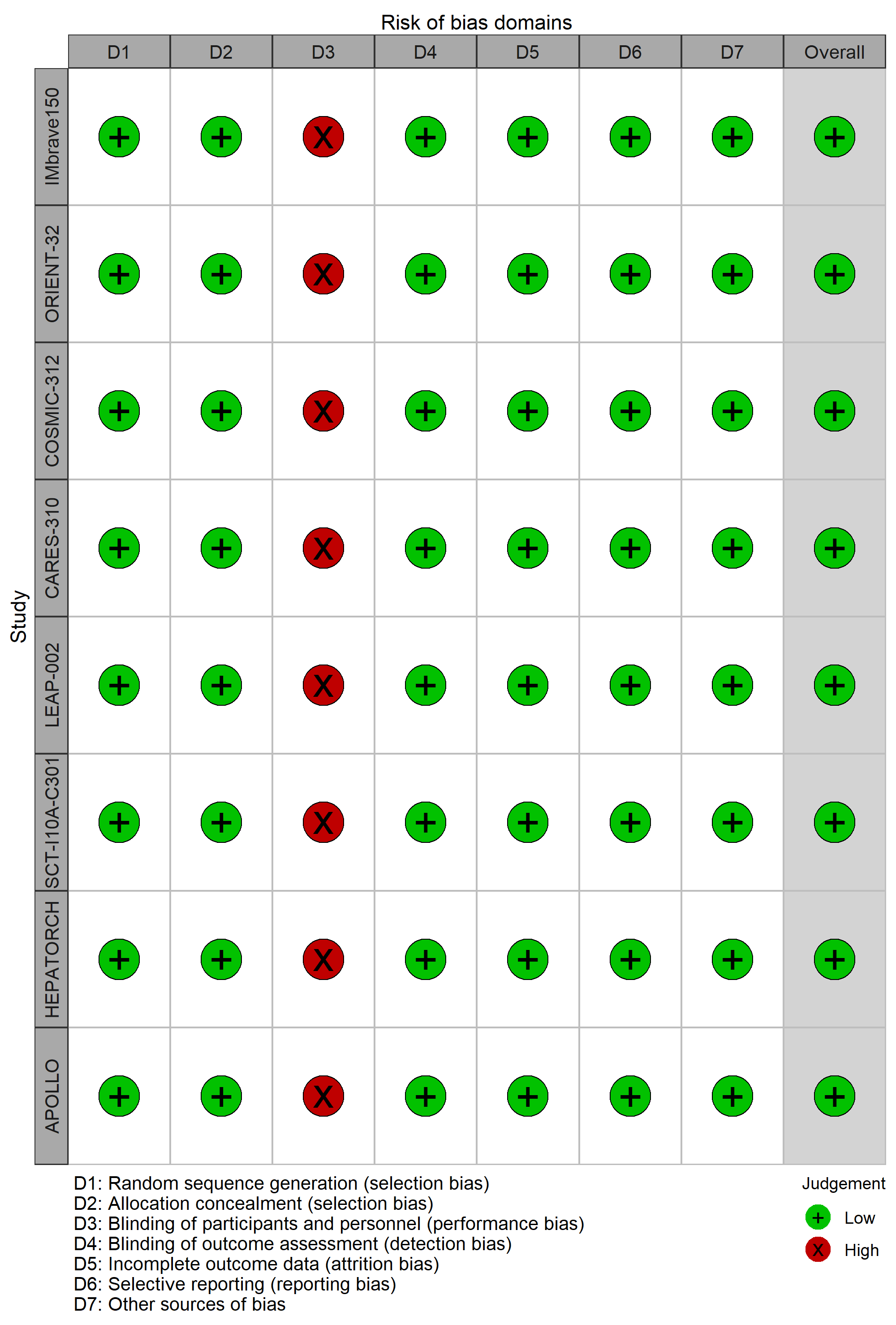
**

**Supplementary Figure 12.** Risk of bias graph.

**
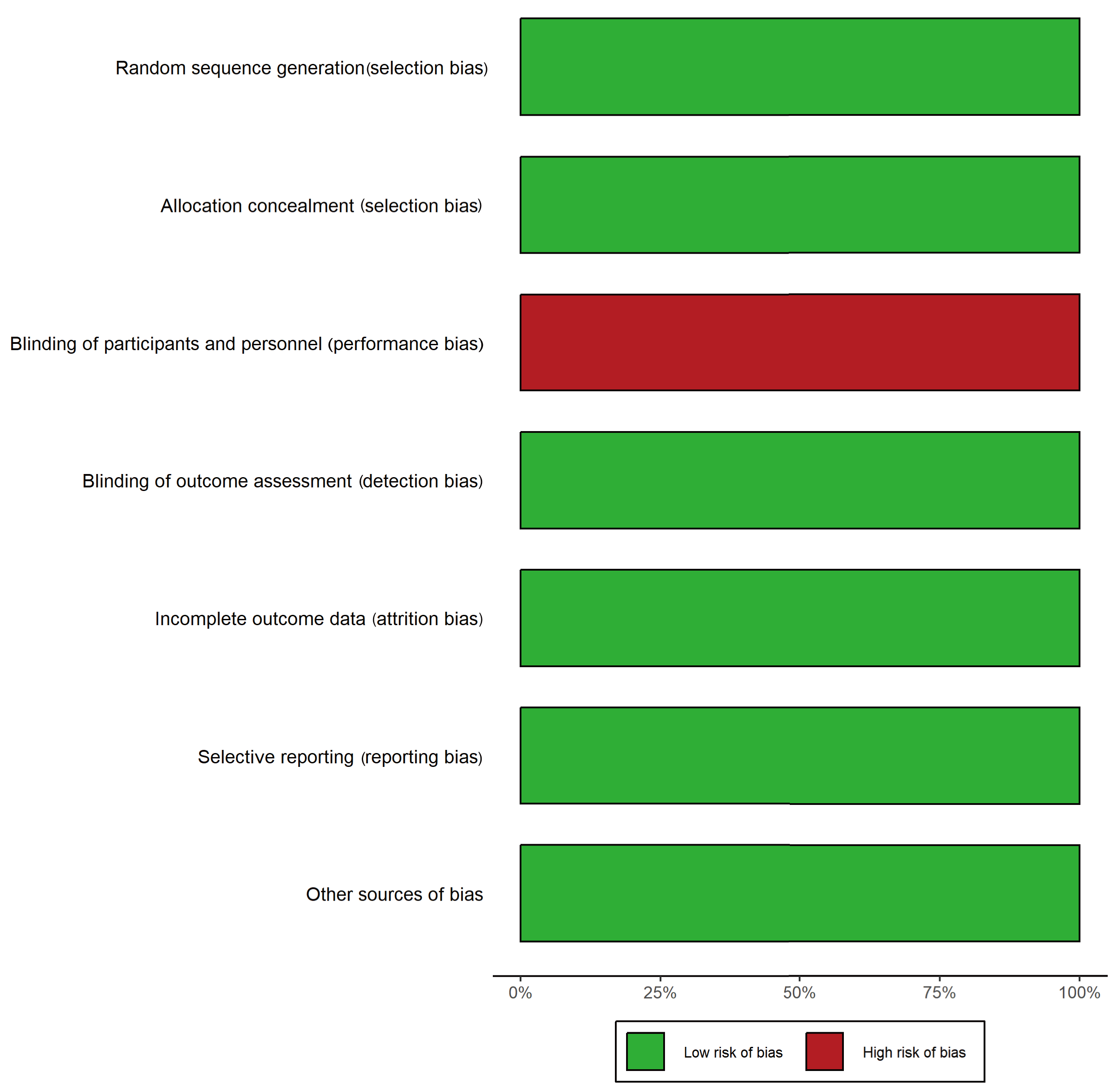
**

**Supplementary Figure 13.** Risk of bias summary.

**
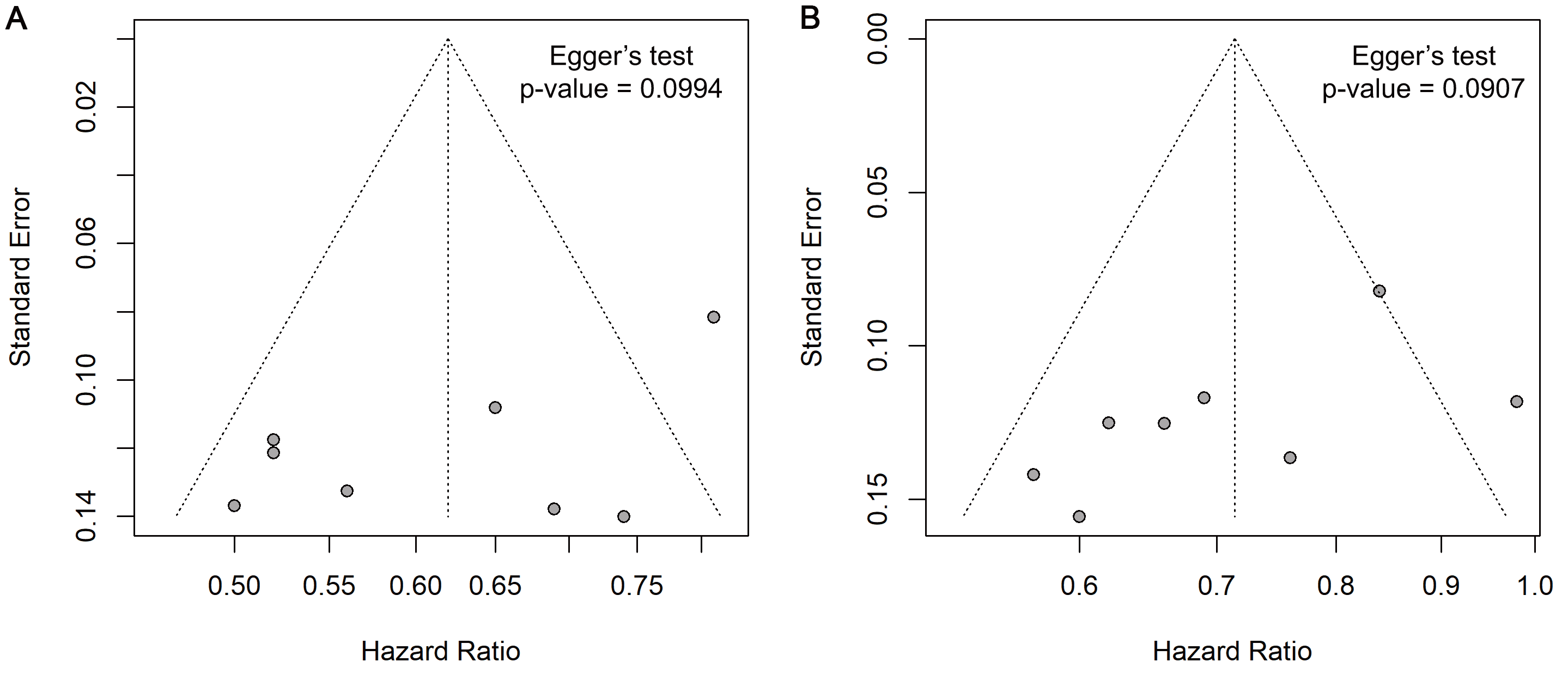
**

**Supplementary Figure 14.** Funnel plots and Egger’s tests for progression-free survival (**A**) and overall survival (**B**).


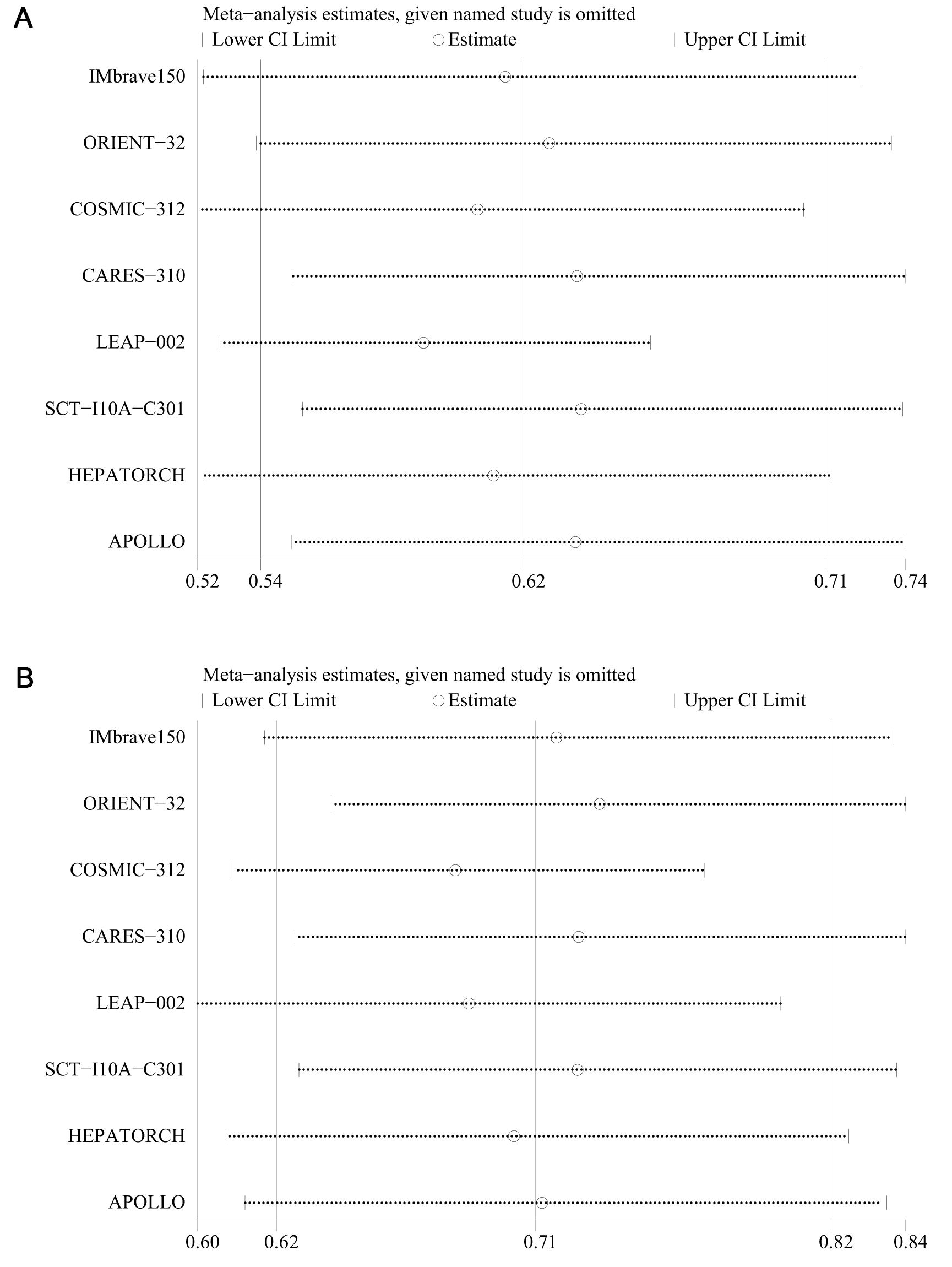


**Supplementary Figure 15.** Sensitivity analyses for included studies on progression-free survival (**A**) and overall survival (**B**) examined by leaving-one-out approach.
